# A molecular phenology scale of grape berry development

**DOI:** 10.1093/hr/uhad048

**Published:** 2023-03-15

**Authors:** Giovanni Battista Tornielli, Marco Sandri, Marianna Fasoli, Alessandra Amato, Mario Pezzotti, Paola Zuccolotto, Sara Zenoni

**Affiliations:** Department of Biotechnology, University of Verona, Strada Le Grazie 15, 37134 Verona, Italy; Department of Biotechnology, University of Verona, Strada Le Grazie 15, 37134 Verona, Italy; Big & Open Data Innovation Laboratory, University of Brescia, C.da S. Chiara 50, 25122 Brescia, Italy; Department of Biotechnology, University of Verona, Strada Le Grazie 15, 37134 Verona, Italy; Department of Biotechnology, University of Verona, Strada Le Grazie 15, 37134 Verona, Italy; Department of Biotechnology, University of Verona, Strada Le Grazie 15, 37134 Verona, Italy; Big & Open Data Innovation Laboratory, University of Brescia, C.da S. Chiara 50, 25122 Brescia, Italy; Department of Biotechnology, University of Verona, Strada Le Grazie 15, 37134 Verona, Italy

## Abstract

Fruit growth and development consist of a continuous succession of physical, biochemical, and physiological changes driven by a genetic program that dynamically responds to environmental cues. Establishing recognizable stages over the whole fruit lifetime represents a fundamental requirement for research and fruit crop cultivation. This is especially relevant in perennial crops like grapevine (*Vitis vinifera* L.) to scale the development of its fruit across genotypes and growing conditions. In this work, molecular-based information from several grape berry transcriptomic datasets was exploited to build a molecular phenology scale (MPhS) and to map the ontogenic development of the fruit. The proposed statistical pipeline consisted of an unsupervised learning procedure yielding an innovative combination of semiparametric, smoothing, and dimensionality reduction tools. The transcriptomic distance between fruit samples was precisely quantified by means of the MPhS that also enabled to highlight the complex dynamics of the transcriptional program over berry development through the calculation of the rate of variation of MPhS stages by time. The MPhS allowed the alignment of time-series fruit samples proving to be a complementary method for mapping the progression of grape berry development with higher detail compared to classic time- or phenotype-based approaches.

## Introduction

The ontogenic development of fleshy fruits entails an ordered sequence of physical, chemical, physiological, and molecular changes that progressively drive the entire organ towards maturation. These processes are rather conserved among fruits of the same species, but the developmental progression may vary due to both genetic and environmental factors. Moreover, developmental expression patterns are extremely dynamic and, especially under fluctuating environmental conditions [1–3], rapid changes may happen within short time windows, challenging the setup of meaningful comparisons to study the fruit response to any factor. In fruit crops, the ontogenic development of the fruit is tracked by adopting phenological scales. These are classification tools that describe seasonal and precisely recognized stages of fruit growth and development based on specific descriptors such as visual/physical traits or easy-to-measure compositional parameters [4, 5]. Phenological scales are widely used in models describing known or hypothetical cause-effect relationships between growth stages progression and environmental driving factors [6–8].

In grapevine (*Vitis vinifera* L.), the fruit phenophases comprise a large dedicated section of the most adopted phenology scales, namely the modified Eichhorn and Lorenz (E-L) [9] and the extended BBCH systems [10], that assign increasing numbers to the main fruit developmental stages from setting to maturity. Grape berry development consists of two phases of growth and lasts up to 150 days [11]. The first phase (also called berry formation or herbaceous/green phase) involves pericarp growth due to rapid cell division and elongation. The second phase (ripening) involves physical and metabolic changes, including softening, skin pigmentation, accumulation of sugars, loss of organic acids, and synthesis of volatile aromas [12]. The onset of ripening (veraison for viticulturists) occurs after a short lag phase, when the seed maturation is complete [11, 13]. Growth stages are defined by the assessment of visual/physical traits, such as color, size, and softness. Only for ripening stages and harvest decision, are compositional parameters such as the sugar concentration of the juice considered. However, the precise definition of developmental stages can be challenging as the fruit traits used for stage description are highly influenced by several factors like genotype, climate, water availability, agronomical practices, and crop load [14–16].

The advent of next-generation sequencing represents an opportunity to exploit the expression kinetics of large sets of genes to stage fruit development and enrich the available classification systems incorporating information at a molecular level [17, 18]. This approach succeeded in developing a transcriptomic aging clock defining the biological age of organisms such as *C. elegans* to an unprecedented accuracy [19] and, in plants, was used to reconstruct the transcriptional ontogeny of single organs and correlate the appearance of morphological characteristics with molecularly defined developmental stages [20]. Moreover, results obtained using model organisms such as Arabidopsis, or annual crops such as rice, revealed seasonal patterns of gene expression controlled by environmental cues [21, 22] and demonstrated that the recent advancement in the methods for gene expression quantification could be exploited to refine phenological stage classification [23].

A large number of transcriptomic studies of grape berry development generated in recent years revealed that the variation of a portion of the fruit transcriptome is conserved across cultivars and growing conditions [13, 24–28], and thus may be utilized to boost the description of the fruit developmental stages with a molecular dimension.

In this work, we used the most informative portion of several grapevine fruit transcriptomic datasets [13, 26] to build a molecular phenology scale (MPhS). The performance of the scale in precisely mapping the progression of fruit development, compared to other classical time- or phenotype-based approaches was assessed. The MPhS was used to reinterpret previously published transcriptomic datasets evidencing its potential to trace and compare fruit developmental stages from different genotypes and growing conditions.

## Results

### Molecular phenology map creation

To create a molecular scale of grapevine berry development we relied on the RNA-sequencing dataset consisting of 219 samples published by Fasoli et al. [13]. Samples were collected from fruit set to full maturity from Cabernet Sauvignon (CS) and Pinot noir (PN) vines every 7–10 days across three years (Table S1). The technological and molecular data, and the related methodologies are reported in the original paper [13].

**Figure 1:**
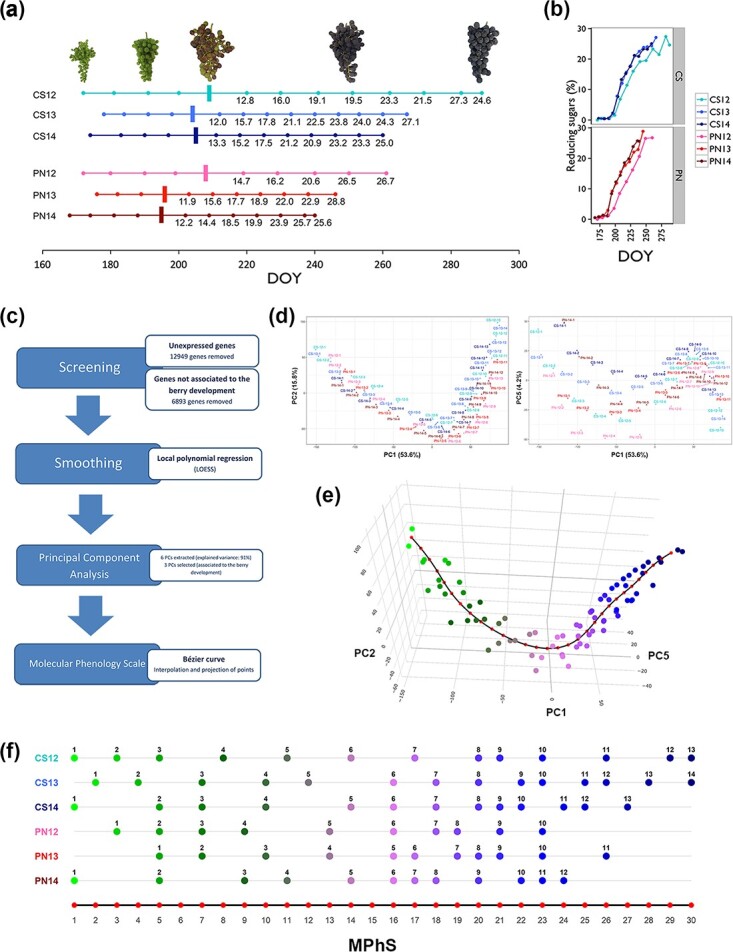
Molecular phenology map creation. (a) Cabernet Sauvignon and Pinot noir time series of berry sample collection. Berries were collected using a randomized block approach to account for intra-vineyard block variability. This resulted in a collection of 219 samples (CS12: 13 time points; CS13: 14; CS14: 13; PN12: 10; PN13: 11; PN14: 12. Each sample was collected in triplicates). Brix values are specified for time points post veraison (marked by vertical bar) along the timeline. Veraison: visually defined as the 50% of all berries - in the bunch beings colored. DOY, day of the year. Images on top show samples depicting five stages of grape cluster development for Cabernet Sauvignon during season 2012. (b) Accumulation trends of reducing sugars (%) by DOY in Cabernet Sauvignon and Pinot noir samples over three years. Plots were generated using R package ggpplot2 version 3.4.1 [29]. DOY, day of the year. (c) Flow chart of the pipeline. The chart represents the four principal steps (blue rectangles) and related details (white rectangles) (d) Samples distribution according to the three selected PCs. PC1 (53.6%) by PC2 (15.8%) (left) and by PC5 (4.2%) (right). (e) Three-dimension scatterplot of the three selected PCs interpolated by the Bézier curve (black line). Red dots along the Bézier curve define a set of 30 evenly spaced molecular stages. Scattered points correspond to the smoothed samples and changing color highlight berry progression from immature to ripe stages. An interactive plot of the curve is accessible at the link https://bodai.unibs.it/grapevine-gea/mphs/. (f) Projection of the smoothed three-year time series of CS and PN to the closest point among the 30 points identified along the Bézier curve. The sample number is reported above each point. Red dots correspond to the 30 steps of the Molecular Phenology Scale.

Vintage variability impacted ripening progression since its onset happened earlier in 2013 and 2014 compared to 2012, which resulted in a substantial advance until the grape technological maturity as monitored by sugar accumulation (Fig. 1a,b). This behavior was more evident in PN, an earlier ripening variety compared to CS.

Fasoli and co-authors looked into the biological functions of the genes partaking in the berry developmental waves of expression, specific or shared by the two genotypes, and expounded on the transcriptome rearrangements associated with the onset of ripening [13], whereas the transcriptomic data was here analyzed using statistical and data mining tools to identify the core set of genes that define the berry development progression (Fig. 1c). The pipeline comprised an initial screening to discard genes exhibiting low and/or noisy expression that reduced the dataset from 29 971 to the whole core set (WCS) of 10 129 genes (Dataset S1). Genes included in the WCS were characterized by consistent expression behaviors during development across genotypes and vintages. The comparison with the transcriptomic dataset of Massonnet et al. [26] was also part of the initial screening to ensure the congruency of gene expression in white and another red skinned grapevine varieties. This step was followed by the application of a local polynomial regression that allowed smoothing the gene expression patterns over the time points and averaging the replicates. We then performed a Principal Component Analysis (PCA) with the data matrix obtained by column-standardization of the smoothed gene expression. We selected Principal Components (PCs) 1 and 2 that best described the general progression of berry development, and PC5 that improved the discrimination of early-stage samples (Fig. 1d), whereas the effect of genotype and vintage (PC3, 4 and 6) was excluded (Fig. S1).

The PC1, 2 and 5 defined a three-dimensional scatter of points, with each point corresponding to an experimental condition (one time point for one cultivar in one year) that were then fitted by one-dimensional space using a Bézier curve. Thirty marks were evenly distributed along the curve to represent steps of what we called Molecular Phenology Scale (MPhS) (Fig. 1e). The points of the 3D scatter were projected onto the MPhS and assigned to the closest among the set of 30 marks, demonstrating that the chronological order of all sampling series was maintained along the MPhS (Fig. 1f), and the core gene set represented a suitable selection for our purpose.

### MPhS offers insights into the ripening stages reached during CS and PN berry development

**Figure 2:**
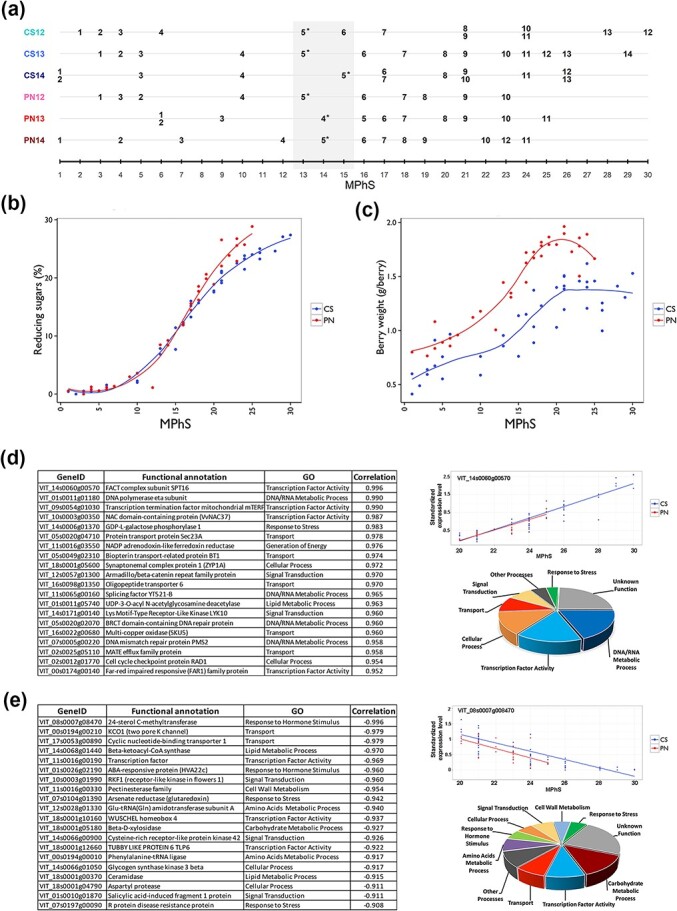
Relationship between Molecular Phenology Scale and time during fruit development. (a) Projection of the non-smoothed three-year time series of CS and PN on the MPhS. Samples collected at veraison phase are marked with asterisks. The light-grey rectangle highlights the MPhS stages corresponding to the veraison transition. (b) Trend of percentage of reducing sugars accumulation by MPhS in CS and PN. Smoothed conditional means function of the R package ggpplot2 version 3.4.1 [29] was used to represent the average of the three years per genotype. (c) Trends of berry weight by MPhS, in CS and PN. Smoothed conditional means function of the R package ggpplot2 version 3.4.1 was used to represent the average of the three years per genotype. (d-e) Top-20 most positively (d, left) and negatively (e, left) correlated genes with the 20-to-30 MPhS stage progression, example of expression trend of the highest positively (d, right upper part) and negatively (e, right upper part) correlated genes and pie chart of the functional category distribution of the 72 positively (d, right lower part) and the 53 negatively (e, right lower part) correlated genes.

The performance of the MPhS was next explored by projecting the non-smoothed three-year time series of CS and PN onto the MPhS. We found that, for the most part, the samples were correctly ordered according to their chronological collection (Fig. 2a and Table S2). Four sample pairs of CS mapped at the same MPhS stages and, two instances each genotype, samples collected at the beginning or at the end of the series showed mapping discrepancy with the scale sequence, suggesting a lower resolution of the MPhS at phases with reduced time-related transcriptomic changes. In fact, the samples were not evenly distributed along the MPhS and some intervals (e.g. between MPhS stage 7 and 12) were poorly represented. To evaluate if the differences in the MPhS resolution could be related to variations in samples uniformity along the development of the fruit (e.g. samples at the initial and final timeframes as opposed to mid-development) we averaged the Coefficients of Variation (CV) of each transcript expression value in the triplicates at each time point (Fig. S2). This analysis showed that sample replicates collected in the 7–14 MPhS window are more consistent than at earlier and later stages. The veraison phase, as defined by the BBCH scale [10], was pinpointed in correspondence to the MPhS window 13–15 for both genotypes, whereas PN always reached sugar maturity at earlier MPhS stage compared to CS.

When exploring the association between MPhS stage and sugar content for PN and CS samples, we observed a strict non-linear relation that clearly varied in the two genotypes, with PN reaching technological maturity at earlier MPhS stages than CS (Fig. 2b). On the other hand, an elevated variability, beyond the clear genotype effect, was observed between MPhS and berry weight, suggesting that the two variables are poorly related (Fig. 2c and Fig. S3). This analysis also evidenced that PN berries lost weight during the last ripening stages, while CS berry weight remained steady.

In order to gain some insights into the biology of late MPhS ripening stages that appeared to be missing in PN, we explored the function of genes highly correlated with the 20-to-30 MPhS stage progression (Fig 2d-e; Fig. S4 and Dataset S2). Among the top-20 positively correlated genes, several transcription factors and genes involved in DNA/RNA metabolic process and transport, were identified, whereas a greater variety of functional categories was found for the top-20 negatively correlated, among which response to hormone stimulus, signal transduction and transcription factor activity (Fig 2d-e; Fig. S4). At a correlation coefficient cutoff >|0.7| (selecting 72 positively and 53 negatively correlated genes; Dataset S2), the functional categories distribution resulted distinctly different between the positively correlated, enriched in the DNA/RNA metabolic process category, and the negatively correlated genes, involved in carbohydrate metabolic process, amino acid metabolic process, response to hormone stimulus and cell wall metabolism (Fig 2d-e). Surprisingly no genes related to secondary metabolism have been identified in either list (Dataset S2). We also calculated the correlation of the selected genes with the 1-to-10 and 10-to-20 MPhS stage progression. This analysis revealed that the genes positively correlated to the 20-to-30 stage progression behaved inconsistently when evaluated in the other intervals. Instead, large part of the genes negatively correlated to the 20-to-30 stage progression confirmed a negative association at previous MPhS intervals (Dataset S2).

### Relationship between MPhS and time

**Figure 3:**
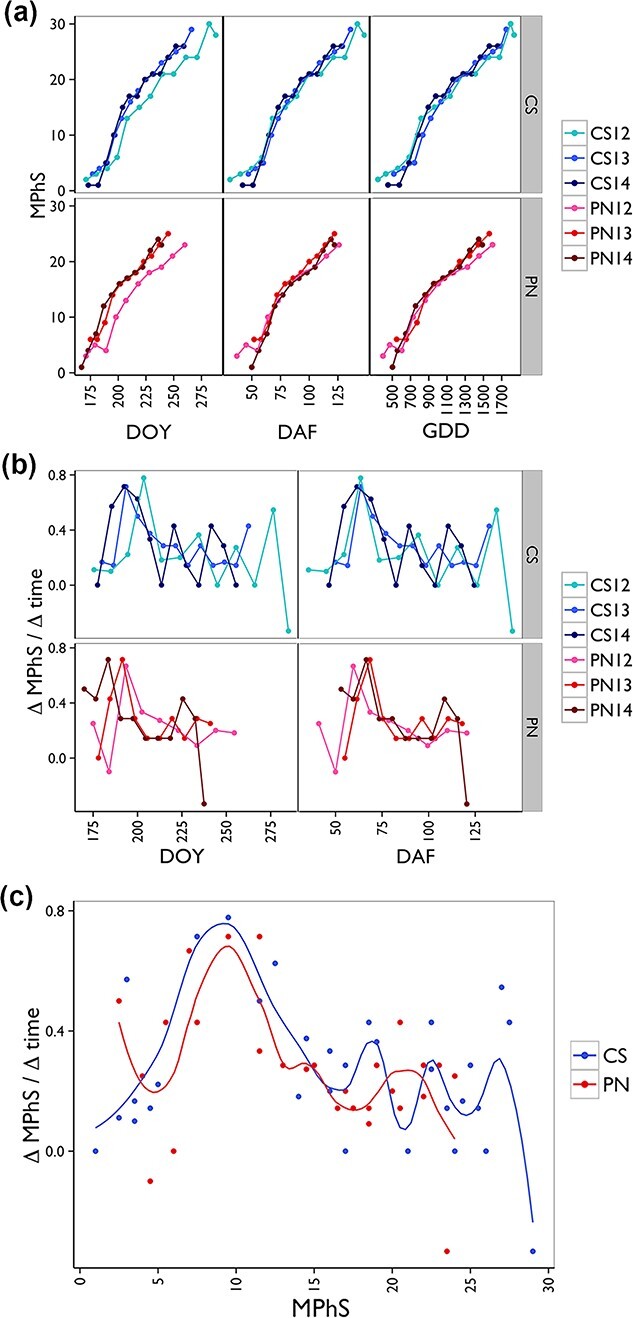
Relationship between Molecular Phenology Scale and time during fruit development. (a) Trends of MPhS stages by day of the year (DOY) (left), day after flowering (DAF) (middle) and Growing Degree Days (GDD) (right) in CS and PN over the three years. (b) Plot of the ΔMPhS/Δtime over DOY (left) and DAF (right). ΔMPhS represents the difference in MPhS between subsequent stages. Δtime represents the difference in days between subsequent stages. Plots were generated using R package ggpplot2 version 3.4.1 [29]. (c) Plot of the ΔMPhS/Δtime over the MPhS. Smoothed conditional means function of the R package ggpplot2 version 3.4.1 was used to represent the average of the three years per genotype.

Plotting the MPhS by the day of the year (DOY) confirmed differences among years and evidenced a transcriptional progression delayed in 2012 in both varieties, whereas the alignment on the phenological flowering phase (days after flowering, DAF) resulted in nearly overlapping curves (Fig. 3a). This representation unraveled common kinetics, reflecting a non-linear relationship between time and MPhS stages with the very first phase characterized by slow transcriptional changes followed by a transient rapid advancement (Fig. 3a). Given the well-known close relation between temperature and phenology, we evaluated whether seasonal temperature regimes could be the driver of the variable MPhS stage progression rate. When the MPhS series were plotted on the heat summation calculated for each season, we found dynamics alike what observed for DAF (Fig. 3a). This suggests that the transcriptome evolution is sometimes faster or slower over the course of fruit development and that such intrinsic variations are not directly related to seasonal temperature regimes.

The transcriptomic scale dynamics were better highlighted by the ratio ΔMPhS/Δtime (an approximation of the derivative of the MPhS curve over time) showing clear fluctuations irrespective of the DOY and was better aligned for each genotype when plotted against the day after flowering (DAF), with a major peak likely associated with the onset of ripening, followed by some minor peaks (Fig. 3b). We further explored the MPhS features by plotting the ΔMPhS/Δtime averaged by year over the MPhS itself, revealing that indeed some MPhS stages are rapidly passed through by developing berries (Fig. 3c). A main increase in rate was reached at stages 9–10 in both genotypes, followed by minor accelerations at stages 17–18, 22–23 and 27–28 in CS and around stage 22 in PN.

### Mapping other transcriptomic samples onto the MPhS

**Figure 4:**
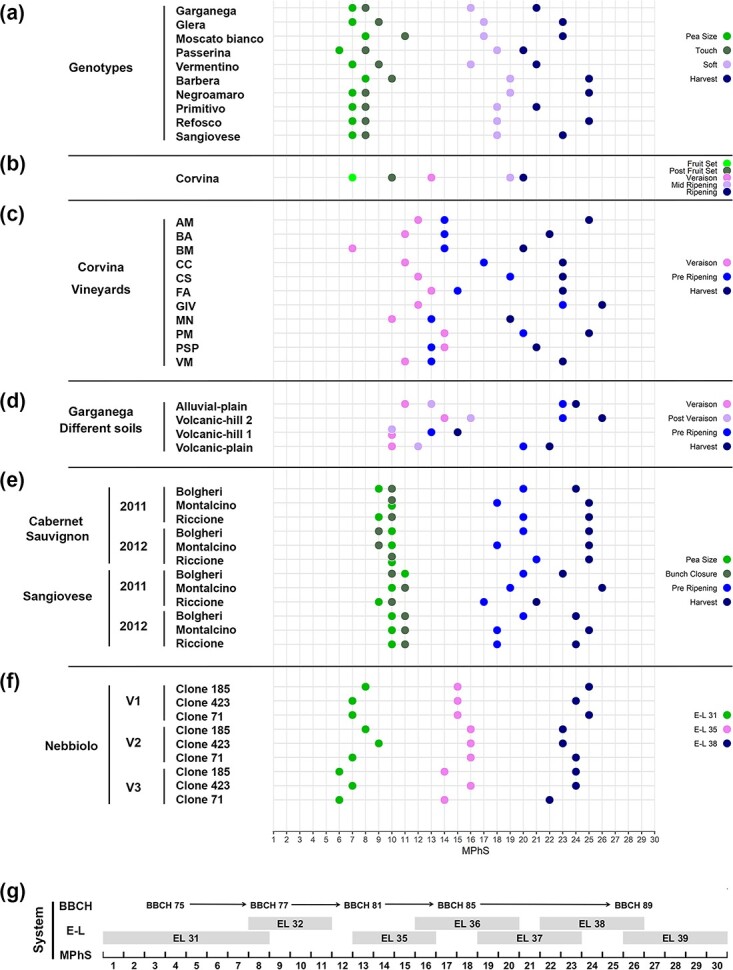
Scaling of previously published berry transcriptomic samples onto the MPhS. (a) Projection of RNA-seq transcriptomic berry samples of ten varieties [26] onto the MPhS. The BBCH scale was followed to collect berry samples at specific phenological stages: Pea Size (BBCH 75), Touch (BBCH 77), Soft (BBCH 85) and Harvest (BBCH 89) during the same season. (b) Projection of microarray transcriptomic berry samples of cv Corvina [25] onto the MPhS. Samples collection was performed at phenological stages defined by the E-L scale: Fruit Set (E-L 29), Post Fruit Set (E-L 32), Veraison (E-L 35), Mid Ripening (E-L 36) and Ripening (E-L 38). (c) Projection of microarray transcriptomic berry samples of cv Corvina collected at three ripening stages (Veraison, Pre Ripening and Harvest) from eleven cultivation sites [24], onto the MPhS. (d) Projection of microarray transcriptomic berry samples of cv Garganega collected at four ripening stages (Veraison, Post Veraison, Pre Ripening and Harvest) from four cultivation sites [30], onto the MPhS. (e) Projection of microarray transcriptomic berry samples of cv Sangiovese and cv Cabernet Sauvignon compared across three growing sites and over two years [31], onto the MPhS. Berries were collected at four developmental stages defined by the BBCH phenological scale: Pea Size (BBCH 75), Bunch Closure (BBCH 79), Pre Ripening (BBCH 83) and Harvest (BBCH 89). (f) Projection of RNA-seq transcriptomic berry samples of three clones of cv Nebbiolo, compared across three different sites [32], onto the MPhS. Samples were collected at three developmental stages defined by the E-L phenological scale: Pea Size (E-L 31), Veraison (E-L 35), and Ripening (E-L 38). We represented seven main stages in the legend: stage 1 (Fruit Set and E-L 29; light green), stage 2 (Pea Size and E-L 31; green), stage 3 (Touch, Post Fruit Set, Bunch Closure and E-L 32; olive green), stage 4 (Veraison and E-L 35; pink), stage 5 (Soft, Mid Ripening, Post Veraison and E-L 36; plum), stage 6 (Pre Ripening and E-L 37; blue) and stage 7 (Harvest, Ripening and E-L 38, midnight blue). (g) Alignment between MPhS and two phenotype-based phenological scales (BBCH and the modified E-L) during berry development.

The performance of the MPhS was then tested on previously published berry transcriptomes (performed by RNAseq or microarray platforms) describing berry development for different varieties and at varying growing conditions. The relative samples were mapped onto the MPhS using the core set of 10 129 genes selected for the scale definition. The RNA-seq dataset from Massonnet et al. [26] provided information in 10 genotypes across four berry phenological stages (BBCH scale) of the same season. When scaled onto the MPhS, the two early stages, Pea Size and Touch, mapped nearby between stages 6 and 11 (Fig. 4a). Later stages appeared less aligned across genotypes, spanning from the white-skin variety Passerina that mapped at MPhS stage 20 at Harvest, to the red-skin Barbera, Negroamaro and Refosco, exhibiting maturity at 25.

We also scaled samples from microarray-based transcriptomic datasets among which the five phenological stages (E-L scale) of the cultivar Corvina [25]. Albeit obtained by a different transcriptomic technology, samples mapped neatly onto the MPhS, with green berry samples projected at stages 7 and 10, Veraison at stage 13, whereas Mid Ripening and Ripening at stages 19 and 20, respectively (Fig. 4b).

A benefit of the molecular scale consists in recalibrating studies that organized sampling on a time-based approach rather than following berry phenology. This was the case of two microarray-based transcriptomic datasets in which berries of the cultivar Corvina were collected at three ripening times from 11 sites [24], whereas for the cultivar Garganega samples were collected at four ripening times from four sites [30]. As expected, datasets projection onto the MPhS revealed a fair misalignment of time points collected from different sites (Fig. 4c,d). The greatest differences were observed for Corvina samples at the second stage ranging from MPhS stages 13 to 23, whereas Garganega berries mapped between MPhS stages 15 and 26 at harvest. Given the availability of technological ripening data we investigated the relation between sugar content and MPhS stage in above-mentioned collections. As expected, samples that could be considered advanced in ripening by a phenotypic assessment (i.e. sugar content) generally matched advanced MPhS stages (Fig. S5). However, the relationship between the two variables was far from close, especially in the Corvina samples collected from different growing sites, indicating that defining the ripening stage by sugar level may not meet the actual physiological ripening stage when the grapes are grown under different environmental conditions.

Mapping the samples from the work of Dal Santo and co-authors [31] that explored the genotype by environment interaction (GxE), revealed that berries of the cultivar Sangiovese reached maturity at different MPhS stages by cultivation site and year, whereas Cabernet Sauvignon samples in comparison appeared much more aligned (Fig. 4e), affirming the marked transcriptomic plasticity of Sangiovese. The strength of the MPhS mapping approach to GxE was also assessed when the genotype component represented different clones, like in the work of Pagliarani et al. [32] entailing RNA-seq berry samples of three clones of the cultivar Nebbiolo, grown in three sites, and collected at three developmental stages. Although samples arranged along the MPhS by E-L phenological classification (Fig. 4f), minor MPhS shifts reflecting the intrinsic transcriptomic plasticity of each clone interacting with the growing site were still appreciable.

The available sampling metadata was then exploited to attempt an alignment between our MPhS and the classical phenotype-based phenological scales (i.e. the modified E-L and BBCH systems) during berry development (Fig. 4g), showing the greater classification detail provided by the MPhS compared to the traditional scales.

### Performance of MPhS based on a reduced core set of genes

**Figure 5:**
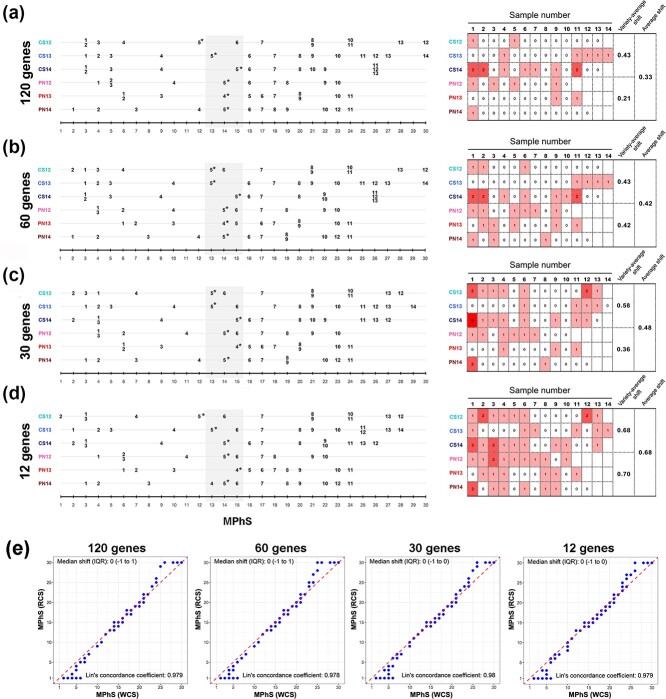
Performance of MPhS based on a reduced core set of genes. Projections of the sample series defined by the progressively reduced core set of genes (a) 120 genes, (b) 60 genes, (c) 30 genes and (d) 12 genes (Dataset S3) onto the MPhS (left) and representation of the average shifts between the distribution of samples defined by reduced core sets versus the distribution defined by the entire core set of genes (10129) (right). The light-grey rectangle highlights the MPhS stages corresponding to the veraison transition. For each sample the shift between the MPhS stage defined by the reduced gene core set and the MPhS stage defined by the entire gene core set was calculated. The overall and by variety average shift are shown per reduced core set of genes. (e) Graphical representation of the agreement between the projection on the MPhS defined using the entire core set of genes and the MPhS defined by reduced core sets of 120, 60, 30 and 12 genes. The red dashed line represents the line of perfect agreement. The plots report the estimated Lin’s concordance coefficient and the median (with interquartile range, IQR) of the shift (difference) between the two scales.

To improve the feasibility of our scale and build it around performing targeted expression analysis on a limited number of genes, we focused on identifying the smallest number of genes necessary to efficiently map berry transcriptomic samples onto the MPhS. Starting from the core set of genes used to create the MPhS, pools of 20, 10, 5 and 2 positive and negative loadings of each of the three PCA components (corresponding to 120, 60, 30 and 12 genes, respectively) were selected based on their absolute correlation value (p-corr) and expression profile. Hierarchical cluster analysis of the top-100 loadings throughout berry development in PN and CS during the three vintages revealed 20 main clusters of gene expression (Fig. S6). The selection of the 120 and 60 genes was based on the loadings with the highest p-corr values, being representative of the most populated clusters. As genes belonging to the same metabolic/developmental process likely co-express, for the more stringent selection of 30 and 12 genes we picked those with high p-corr value belonging to different clusters to maximize the information potential thus avoiding redundancy (Dataset S3). We projected PN and CS sample series defined by the reduced core sets (RCSs) and compared them with their projection based on the WCS. This analysis highlighted that the progressive reduction of the core gene set from 120 to 12 did not substantially impacted the samples order (Fig. 5a-d left). In order to evaluate any difference in performance between the projections we calculated the average of the absolute shift values, the quartile shift using non absolute values and the Lin’s concordance coefficient [33] (Fig. 5). In both varieties, the average shift values showed only a slight increase with the progressive reduction of the number of selected genes in RCSs and reached the highest value of 0.7 in PN with the RCS of 12 genes. Accordingly, quartile analysis and Lin’s coefficients (values between 0.95 to 0.99) indicated a substantial concordance [33] among MPhS stages obtained by using the WCS, and MPhS stages obtained using the RCSs from 120 to 12 genes (Fig. 5e).

In order to further evaluate the stability of RCSs across different genotypes and growing conditions we projected on the MPhS samples of the abovementioned transcriptomic work of Massonnet et al. [26] and Dal Santo et al. [24, 30], using the RCSs of 120 and 30 genes (Fig. S7). Quartile analysis showed a general slightly higher deviation compared to PN and CS, with Lin’s coefficients indicating moderate to substantial concordance (values between 0.9–0.99), except for RCS of 30 genes in Garganega (0.89).

## Discussion

Assembling meaningful comparisons between fruits characterized by different developmental rates or seasonal developmental shifts is a major limitation in studying the biological mechanisms underpinning the seamless developmental progression that leads the fruit to maturation. The opportunity of refining the existing development classification systems by integrating phenotype with molecular-based information has been explored in model organisms or annual crops [19, 21, 23]. The transcriptomic analysis of immature maize tassels and sorghum panicles throughout development enabled the reconstruction of their transcriptional ontogeny and correlated the emergence of species-specific morphological characteristics of these organs to developmental stages defined at molecular level [20]. Despite omics technologies being widely applied to unveil and describe in detail the ongoing developmental changes in fleshy fruits from many species [13, 34–36], the molecular information has rarely been exploited to attempt at scaling the fruit ontogeny. In grapevine, Dai et al. [37] demonstrated that metabolite profiling can be used in PCAs to define development trajectories for berries, whereas Wang et al. [38] showed that the expression trend of a small number of genes involved in grapevine flower and berry development was associated to some specific phenological stages.

In this study, we selected about ten thousand genes from several transcriptomic datasets for their consistent expression throughout berry development regardless genotype and season [13, 26] and built a molecular phenology scale to map the ontogenetic development of the fruit with high precision and to align berry development of different grapes.

The training dataset [13] included the transcriptomic samples from two red varieties collected at weekly intervals, that to date represents the most exhaustive survey of the molecular changes during grape berry development. Although the training dataset is based on red varieties only, we are confident that the MPhS could represent the molecular dynamics of both red and white berry varieties because: (i) it was shown [26] that the majority of the transcriptomic changes are shared by the developing berries of both red and white varieties and that (ii) the final steps of the transcriptomic route crossed by the developing berries are covered only by the red berries; (iii) the WCS of genes used to build the scale were selected for the consistency of their expression among transcriptomic time-series of both red and white berry varieties. The biological meaning of the shorter transcriptomic route observed in white berries has been thoroughly discussed by Massonnet et al. [26].

The proposed statistical pipeline consisted of an unsupervised learning procedure yielding an innovative combination of semiparametric, smoothing and dimensionality reduction tools. In particular the most important elements of novelty that differentiate our method from similar approaches are: (i) computing the Principal Components on smoothed data, thanks to the high number of observed timepoints that allowed to obtain more stable estimates than those made on raw data; (ii) the exceptional power of the Principal Components in summarizing different characteristics of the data (berry variety, vintage, stage) that allowed to precisely select genes involved in berry development and rule out those impacted by genotype and vintage without performing any preliminary gene selection step; (iii) relying on three Principal Components to account for the ripening process and the use of a Bézier curve in a 3D space to estimate the Molecular Phenology Scale. Time information was exploited by only considering timepoint succession disregarding their distance so the MPhS units are not time but ideal steps of the berry development, which can take longer or shorter, providing the flexibility of accounting for a multiplicity of factors.

When projecting onto the MPhS the sample series used for its own construction, the distribution was largely by the time of collection during the season, with very few overlaps or inversions. These cases were mainly concentrated at the initial and final stages of the scale suggesting that berry transcriptome evolution right after fruit set and near maturity is slower than during the central time frame, therefore samples collected at two consecutive weeks could exhibit very similar transcriptomes. Another cause for the MPhS lower resolution early and late in development may be represented by higher levels of within-bunch, bunch-to-bunch or vine-to-vine variation, reflected by increased variability in the average CV of samples mapping at these stages. This may have naturally diminished the outline of the transcriptional profiles at early and late development compared to those from the more homogenous samples mapping in the central part of the MPhS. Indeed, the uneven distribution of samples along the scale mirrored the complex dynamics of berry transcriptome over development revealing that some MPhS stages are more rapidly passed through than other during fruit development. For both varieties the highest rate of variation of the MPhS stage by time (represented by a major peak) was recorded well prior the assessed veraison stage, confirming that the ripening transcriptional program is established one-to-two weeks before berry phenotypic changes can be visually appreciated [13, 39, 40]. Following the onset of ripening, minor peaks were present towards maturity suggesting that the ripening progression is discontinuous at transcriptional level. It is well known that seasonal temperature regimes greatly affect the developmental progression of the berry. However, the MPhS stage-by-time fluctuations do not appear to be caused by interactions with the environment as the statistical pipeline was designed to screen out the vintage component and any erratic variation of gene expression.

An application of the MPhS could be aligning samples to highlight shifts of fruit development driven by factors like genotype and environment as well as precisely quantifying their transcriptomic distance. This could represent a start from which to pinpoint the specific contribution of any variance in fruit development and the cultivation environment to the overall plasticity. Once fruit samples are aligned by MPhS stage, comparing differentially expressed genes could unveil responses uniquely related to the growing conditions. Projecting RNA-seq and microarray transcriptomic samples [24–26, 30–32] onto the MPhS indeed highlighted phenological differences and provided the basis to understand why samples originally assigned to similar phenological stage mapped relatively apart along the MPhS. Nevertheless, the ability of MPhS to discriminate samples collected with a considerable separation in time was not fully confirmed in certain dataset/time series. This may be related to specific raw data processing and procedures adopted in each study, prompting the need of future investigation and development of broader normalization protocols to improve the performance of the MPhS. Therefore, at this stage, unless recomputed with the same bioinformatic pipeline, the direct comparison between transcriptomic samples obtained in different studies should be done with cautious.

Regarding the classification of ripening stage, the molecular scale represents an innovative approach over the traditional analytical methods (i.e. total soluble solids or reducing sugars percentage in the grape juice) that, although rapid, do not always represent a reliable indicator of the berry physiological ripening stage. In fact, the great influence of genetics, climate, and agronomic factors on sugar accumulation dynamics in the fruit can lead to a partial uncoupling from other ripening technological parameters, like organic acids and skin pigment content [14–16]. We found a close relation between MPhS stage and berry sugar level at initial stages of maturation in both cultivars, that diverged at late maturation likely reflecting the different length of ripening characterizing the two genotypes and indicating that CS berries reached the target level of soluble solids at an advanced MPhS stage compared to PN. The early achievement of technological maturity in PN is further pointed out by the berry weight decrease in the late developmental stages showing that the final increase in sugar was largely determined by increased solute concentration due to water loss rather than active import through the pedicel. The linkage between MPhS stage and sugars weakened when a single genotype was grown in different environments evidencing that similar sugar levels can match different steps in the ripening transcriptomic program (Fig. S5). Transcriptomic differences in samples defined at the same ripening stage by sugar level have already been shown in grapes grown in different locations, confirming that the sugar level in a berry is an unreliable marker of berry maturity when comparing grapes from different climates, and suggesting that considering the abundance of key transcripts may better fulfill the role of marking ripening progression [41].

Interestingly, our investigation of the molecular events associated with the last MPhS stages revealed an activation of genes mainly involved in nucleic acid metabolism and transcriptional activity as already highlighted in Fasoli et al. [13]. The biological meaning of such finding is not obvious. Ghan et al. [42] showed that nucleic acid metabolism, chromosome organization and epigenetic regulation represent the main functional categories of genes whose expression is significantly modified in the late stages of ripening of 7 grape varieties. The authors observed that gene networks falling into such categories appeared intimately connected to the core circadian clock gene subnetwork, suggesting that the day length decrease associated with advanced berry ripening and the progression of the season may drive these molecular events, possibly reflecting senescence-related cellular processes. The later harvest of CS than PN grapes may account for the fact that these events are mainly experienced by CS, which then reached more advanced MPhS stages. Moreover, these molecular events may not necessarily have an impact on technological/compositional berry parameters easily detectable by common analytical approaches, further supporting the use of the molecular information for a more precise definition of the fruit developmental stage.

Based upon the metadata available for the datasets mapped onto our scale, we attempted to approximate the 1–30 MPhS stages to those specified in the classical grapevine fruit phenology scales and we thus attested that the MPhS permits greater definition of the phenophases respect to the classical scales [9, 10] that cover longer stretches along berry development (Fig. 4g).

We have also explored the performance of MPhS scale based on a reduced set of genes in different datasets, showing that the number of expression signals necessary for mapping samples can be drastically reduced to a few dozen without substantial loss of precision. Small subsets of (120, 60, 30, 12) genes were identified among PCA loadings and combined to build different versions of the MPhS. While all these estimates showed a good agreement with the MPhS estimated using all genes, the choice of these subset of genes could be further refined to improve the RCS-based MPhS performance. Despite the increasing use of genome-wide approaches, the definition of the molecular phenology stage of a fruit sample by only performing a small number of targeted gene expression analysis may represent a low-cost solution in all those cases where a transcriptomic approach is not an option. Alternatively, this approach could be used to select those samples to be subjected to the transcriptomic analysis from a wide collection of berry samples. The expression analysis of the proposed reduced set of genes, for example by PCR array cards, have the potential to define the developmental berry stage in an easier and faster way compared to omic approaches. The proposed scale paves the way for the development of tools that aspire to predict the phenological stage of the fruit in various climate conditions, such as models that can account for temperature and other environment clues. The quality of these tools will benefit from the combination of various modeling techniques (molecular, metabolite, physical, visual levels), providing that great coordination and knowledge-transfer between modelers, biologists and growers will be established.

The proposed pipeline could be potentially extended and successfully applied to other fruit species, provided they have some basic requirements: (i) a relatively frequent sampling covering the time-series transcriptomic changes during fruit development with high detail; (ii) the availability of expression data from diverse growing conditions and genotypes, allowing the phenology scale to be representative of the general development of the fruit of the species; (iii) a reliable and well annotated reference genome to compute the expression data. The existence of these conditions ensured the successful implementation of our MPhS for the grapevine berry phenological classification, that we foresee will help coping with challenges such as those raised by climate change, allowing the precise mapping of the berry developmental progression.

## Materials and methods

### Data mining process

We used the data described in Fasoli et al. [13] (*dataset A*) and set the following variables:

- Cultivar [Cabernet Sauvignon, Pinot Noir]
- Vintage [2012, 2013, 2014]
- Timepoint [from 1 to 10–14 (different combinations Cultivar x Vintage have different final Timepoints)]

*C*x*V* denotes the six possible combinations Cultivar x Vintage and the term *experimental condition* represents each of the 73 possible combinations Cultivar x Vintage x Timepoint.

Genetic Variables:

- Gene Expression [RNAseq platform, raw RPKM]

${x}_{jri}$ denotes the expression of gene *i* (*i* = 1, 2, . . ., 29 971) in the *j*th experimental condition (*j* = 1, 2, . . ., 73) for the *r*th replicate (*r* = 1, 2, 3); the subscript *h* represents the combination *C*x*V* (*h* = 1, 2, . . ., 6). ${J}_h$ denotes the set of experimental conditions corresponding to *C*x*V = h*.

In addition, ${m}_{ji}$ indicates the average expression (over the three replicates) of gene *i* in the *j*th experimental condition

\begin{align*} {m}_{ji}=\frac{1}{3}{\sum}_{r=1}^3{x}_{jri}\end{align*}

and ${m}_{hi}$ the average expression (over the replicated and the timepoints) of gene *i* in the *h*th *C*x*V* combination

\begin{align*} {m}_{hi}=\frac{1}{3\left|{J}_h\right|}{\sum}_{r=1}^3{\sum}_{j\in {J}_h}{x}_{jri} \end{align*}

where $\left|\cdot \right|$ denotes cardinality of a set.

The expression profiles of each gene were displayed using a three-panel graphical representation (Fig. S8), with one graph for each vintage.

The data mining process comprises four steps (Fig. S9):

**Step 1: Screening.**

We removed 19 842 genes from the 29 971 in the grapevine transcriptome exhibiting uninteresting profiles (i.e. no expression in some experimental conditions or expression not associated to berry development; Fig. S10) based on the criteria summarized in Table S3. To improve the selection, this screening step exploited the information of an additional dataset (*dataset B*), composed of 10 cultivars, observed for 4 timepoints during fruit development in a single vintage [26]. The resulting final set (denoted as *F*) comprised 10129 genes deserving further statistical analysis.

**Step 2: Smoothing.**

Smoothing was applied to the data matrix (219 x 10 129) containing the expressions ${x}_{jri}$ with $i\in F$. For each gene *i* and for each *C*x*V* combination *h*, we estimated smoothed values ${\overline{x}}_{ji}$ by means of a polynomial local regression method called LOESS, introduced by Cleveland [43] and Cleveland and Devlin [44]. LOESS fits a low-degree polynomial to a subset of the data in the neighborhood of each observation of the dataset. In other words, simple parametric models are fitted to localized subsets of the data, aiming at obtaining a smooth curve. The polynomial parameters are estimated by a weighted least squares method, where a higher weight is given to points closer to the observation whose outcome is being calculated. The fraction of the total number of data points that were used in each local fit is determined by the smoothing parameter, usually denoted by α.

In the context of this application, for a gene *i*, the single observation was the expression of replicate *r* of a given cultivar, in a given vintage, at a given timepoint. The neighborhood was composed of all the expressions of gene *i*, for the same cultivar and in the same vintage, in the nearby timepoints, with higher weights to the closest ones. We used polynomials of degree 2 and set α = 0.75. The outcome ${\overline{x}}_{ji}$ for the experimental condition $j\in {J}_h$ was then calculated by evaluating the local polynomial at the timepoint characterizing the experimental condition.

For each gene, a three-panel graphical representation could be visualized, analogous to that presented in Fig. S8, where, in each panel, the line shows the pattern of the smoothed gene expression ${\overline{x}}_{ji}$ (Fig. S11).

**Step 3: Principal Component Analysis.**

This step was applied to the data matrix (73 x 10 129) containing the smoothed expressions ${\overline{x}}_{ji}$ with $i \in F$ . We performed PCA [45] on the standardized data matrix and extracted six PCs accounting for a 91% explained variance. Standardization was inherent to PCA, so it was performed on the overall matrix whose 73 rows are all the possible combinations Cultivar x Vintage x Timepoint. This allows to highlight correlations between genes regardless their level and, at the same time, to identify genotype-specific (PC3/PC6), vintage-specific (PC4) and stage-specific (PC1, PC2, PC5) principal components. The interpretation of the PCs was based on the graphical representations in Fig. S12, where the pattern of each PC was plotted against the timepoints, with lines corresponding to the different *C*x*V* combinations.

**Step 4: Molecular Phenology Scale definition.**

This step concerned the data matrix (73 x 3) containing the values of PC1, PC2 and PC5 for each experimental condition, which can be represented as a scatter of points in a three-dimensional Euclidean space, with the implicit additional information of a timeline expressed by the timepoints and the calendar day. The scatter of points was interpolated by means of a Bézier curve [46], which nowadays is employed in several applications, especially in the field of computer graphics. A Bézier curve aims at fitting points with a smooth curve completely contained within the convex hull of a set of *k* control points (*k* is called the curve’s order): the first and last control points are the end points of the curve, whereas the curve does not pass through the intermediate ones (if any), which define orientation and shape.

In the context of our application, we set *k* = 5 and obtained the curve in Fig. S13. An interactive plot of the curve is accessible at the link https://bodai.unibs.it/grapevine-gea/mphs/. We projected the scatter on the Bézier curve by assigning each point of the scatter to the closest point among a set of 30 evenly spaced points identified along the Bézier curve. We represented the curve as a linear graduation, called the Molecular Phenology Scale. Each experimental condition was assigned to one of the 30 marks and the Molecular Phenology Scale was represented for each *C*x*V* combination *h*, thus showing on the map only the experimental conditions $j\in {J}_h$.

### Projection of transcriptomic data on the MPhS

In this section we describe the procedure to project observations coming from different case studies onto the Molecular Phenology Scale. We used the matrix *A* of the eigenvectors of the three selected Principal Components used to build the Molecular Phenology Scale.

Let ${Z}_{obs}{=}\left\{{z}_{ji}\right\}$ be a $\left|{J}_{obs}\right|$ x $\left|{I}_{obs}\right|$ data matrix containing the observed expression levels of a set ${I}_{obs}$ of genes for a set ${J}_{obs}$ of experimental conditions, where $j=1,2,\cdots, \left|{J}_{obs}\right|$ and $i=1,2,\cdots, \left|{I}_{obs}\right|$. The matrix ${Z}_{obs}$ was column standardized. We consider two cases:

1.
$F\subseteq {I}_{obs}$ (recall that *F* is the set of 10 129 selected genes after the screening step).

In this case we obtained the matrix ${\overset{\sim }{Z}}_{obs}=\left\{{z}_{ji}\right\}$ ($j=1,2,\cdots, \left|{J}_{obs}\right|$ and $i=1,2,\cdots, 10129$) by removing from ${Z}_{obs}$ the columns not belonging to *F*. Using ${Y}_{obs}={\overset{\sim }{Z}}_{obs}\cdot A$ we obtained the estimates of the three Principal Component values for the observed values of the $\left|{J}_{obs}\right|$ experimental conditions;

2)
${I}_{obs}\subset F$ .

This case typically occurs when a limited number of genes is observed, for example for cost reasons. When this is the case, it is recommended to observe genes highly correlated to the Principal Components, that should help obtaining good estimates in spite of the scarcity of data.

Starting from the matrix ${Z}_{obs}$ containing the observations of a limited number of genes $\left|{I}_{obs}\right|$, we obtained the estimates of the Principal Component values for the observed values of the $\left|{J}_{obs}\right|$ experimental conditions as follows:

- we computed the average expression of the $\left|{I}_{obs}\right|$ genes positively and negatively correlated to each Principal Component;
- for Principal Component *q*, we imputed the missing data (non-observed expressions for genes in *F*) with the respective positive or negative average, according to the sign of the corresponding eigenvector coefficient;
- let this imputed $\left|{J}_{obs}\right|\times 10129$ matrix be denoted with ${Z}_{obs,q}$, we obtained the estimates of Principal Component *q* as ${Y}_{obs,q}={Z}_{obs,q}\cdot {A}_q$, where ${A}_q$ was the *q*-th column of *A*.

Once the estimates of the three Principal Component values were obtained, we plotted the estimated three-dimensional scatter. This cloud of points was then projected onto the Bézier curve estimated in step 4 by assigning each point to its closest marker among the set of 30 evenly spaced points previously identified along the curve.

### Selection of transcripts highly correlated with the 20 to 30 MPhS stage progression

We used the data described in Fasoli et al. [13] (*dataset A*) and set the following variables:

- Cultivar [Cabernet Sauvignon, Pinot Noir]
- Vintage [2012, 2013, 2014]
- Molecular Phenology Scale (MPhS)

We will denote *CxV* the 6 possible combinations Cultivar x Vintage and we will use the term experimental condition to denote each of the 73 possible combinations Cultivar x Vintage x MPhS.

Genetic Variables:

Gene Expression level

We denote with ${x}_{jri}$ the expression of gene *i* (*i* = 1, 2, . . ., 29 971) in the *j*-th experimental condition (*j* = 1, 2, . . ., 73) for the *r*-th replicate (*r* = 1, 2, 3); we use the subscript *h* to represent the combination *CxV* (*h* = 1, 2, . . ., 6). ${J}_h$ denotes the set of experimental conditions corresponding to *CxV* = *h*.

In addition, ${m}_{ji}$ denotes the average expression (over the three replicates) of gene *i* in the *j*-th experimental condition

\begin{align*} {m}_{ji}=\frac{1}{3}{\sum}_{r=1}^3{x}_{jri} \end{align*}

and ${m}_{hi}$the average expression (over the replicates and the MPhS) of gene *i* in the *h*-th *CxV* combination

\begin{align*} {m}_{hi}=\frac{1}{3\left|{J}_h\right|}{\sum}_{r=1}^3{\sum}_{j\in {J}_h}{x}_{jri} \end{align*}

where $\left|\cdot \right|$ denotes cardinality of a set.

We selected the subset of experimental conditions characterized by MPhS ≥20 (the last portion of the MPhS) and, for each gene, we applied the part of the data mining procedure proposed in Dal Santo et al. [31] allowing to evaluate - by means of an advanced machine learning algorithm, the Gradient Boosting Machine (GBM, proposed by Friedman [47]) - to what extent each variable (cultivar, vintage, and MPhS) affects the gene expression patterns. To this aim, Variable Importance Measures (*VIMs*) are computed, a nonparametric tool able to describe the impact of variables on the gene expression, taking into account the presence of possible, even complex, interactions among the variables themselves, with a multivariable approach. In this case study, for each gene we computed 3 *VIMs*, related to the variables Cultivar, Year and MPhS, that will be denoted *VIMc, VIMy,* and *VIMs*, respectively. In order to select those genes whose expression pattern is strongly associated with the MPhS and is only mildly affected by the other two factors, we set the following criterion: let ${q}_c^{10},{q}_y^{20}, \textrm
{and}\ {q}_s^{20}$ be respectively the 10^th^, the 20^th^ and the 20^th^ quantiles of *VIMc, VIMy* and *VIMs*, respectively; we selected those genes having $VIMc\le {q}_c^{10}, VIMy\le {q}_y^{20}$ and $ VIMs\ge {q}_s^{20}$.

In order to confirm the goodness of the choice, we computed the Spearman correlation between MPhS and the standardized expression level. All the selected signals exhibit high values of the Spearman correlation which means that strong (linear or nonlinear) monotonic associations are present. *VIMs* estimated by GBM are able to detect more complex (monotonic or nonmonotonic) nonlinear relationships and interactions between covariates, that can be present even when the Spearman correlation is low.

### Selection of transcripts for the reduced core set-based scale

We investigated four scenarios in case 2 (${I}_{obs}\subset F$), characterized by different subsets of genes with |I__obs_| = 120, 60, 30, 12, respectively. For the selection of 120, 60, 30, 12 genes, the expression profile of the top-100 loadings characterized by the highest and lowest correlation values with the three dimensions of the PCA were hierarchically clustered based on average Pearson’s distance metric (Tmev 4.3 software). We selected genes from each of the 20 different clusters. Genes with the highest p-corr value within a cluster were preferred. For the PC5 the selection was based only on the p-corr values (and not on their cluster membership) given their general low levels.

The concordance between two MPhSs, one calculated using all genes and one using a subset of genes, was estimated by Lin’s concordance coefficient [48]. It ranges from −1 to 1, with perfect agreement at 1. According to McBride [33], we used the following interpretation for the coefficient values: < 0.90: poor; 0.90 to 0.95: moderate; 0.95 to 0.99: substantial; > 0.99 almost perfect.

## Supplementary Material

Web_Material_uhad048

## Data Availability

The expression data used for the creation of MPhS was retrieved from Fasoli et al. [13] (data accession number GSE98923), whereas the berry transcriptomes projected onto the MPhS referred to: Massonnet et al. [26] (data accession number GSE62744 and GSE62745); Fasoli et al. [25] (data accession number GSE36128), Dal Santo et al. [24] (data accession number GSE41633); Dal Santo et al. [30] (data accession number GSE75565); Dal Santo et al. [31] (data accession number GSE97578); Pagliarani et al. [32] (data accession number GSE116238).
