# Supplementary material for: A molecular phenology scale of grape berry development
Source: Hortic Res. 2023 Mar 15;10(5):uhad048. doi: 10.1093/hr/uhad048 (PMC10541565; doi:10.1093/hr/uhad048)
Supplement: Web_Material_uhad048 [file web_material_uhad048.zip › Supplementary Material.pdf]

Supplementary Information

Table S1

Description of the sample collection of the Pinot noir and Cabernet Sauvignon dataset from Fasoli et al. (2018)

Sample ID	Variety	Year	Time point	Days After Veraison (DAV)	Day Of the Year (DOY)	Days After Flowering (DAF)	Growing Degree Days (GDD)	Reducing sugars (%)	Berry weight (g/berry)
PN12_0	Pinot noir	2012	1	-36	171	37	398	0.00	0.76
PN12_1	Pinot noir	2012	2	-28	179	45	472	0.52	0.89
PN12_2	Pinot noir	2012	3	-18	189	55	604	1.23	1.08
PN12_3	Pinot noir	2012	4	-9	198	64	729	3.58	1.18
PN12_4	Pinot noir	2012	5	0	207	73	860	8.47	1.44
PN12_5	Pinot noir	2012	6	11	218	84	1004	12.33	1.72
PN12_6	Pinot noir	2012	7	21	228	94	1173	16.19	1.69
PN12_7	Pinot noir	2012	8	32	239	105	1325	20.62	1.86
PN12_8	Pinot noir	2012	9	42	249	115	1451	26.54	1.90
PN12_9	Pinot noir	2012	10	53	260	126	1600	26.75	1.78
CS12_0	Cabernet Sauvignon	2012	1	-37	171	31	343	0.00	0.49
CS12_1	Cabernet Sauvignon	2012	2	-28	180	40	429	0.51	0.64
CS12_2	Cabernet Sauvignon	2012	3	-18	190	50	563	0.45	0.91
CS12_3	Cabernet Sauvignon	2012	4	-9	199	59	686	1.55	0.97
CS12_4	Cabernet Sauvignon	2012	5	0	208	68	817	6.83	0.95
CS12_5	Cabernet Sauvignon	2012	6	11	219	79	963	11.42	1.14
CS12_6	Cabernet Sauvignon	2012	7	21	229	89	1133	16.00	1.37
CS12_7	Cabernet Sauvignon	2012	8	32	240	100	1282	19.14	1.50
CS12_8	Cabernet Sauvignon	2012	9	42	250	110	1409	19.52	1.38
CS12_9	Cabernet Sauvignon	2012	10	53	261	121	1556	23.29	1.62
CS12_10	Cabernet Sauvignon	2012	11	63	271	131	1676	21.47	1.45
CS12_11	Cabernet Sauvignon	2012	12	74	282	142	1798	27.38	1.53
CS12_12	Cabernet Sauvignon	2012	13	80	288	148	1836	24.61	1.41
PN13_0	Pinot noir	2013	1	-20	175	52	548	0.79	0.86
PN13_1	Pinot noir	2013	2	-14	181	58	652	1.17	0.93
PN13_2	Pinot noir	2013	3	-7	188	65	771	2.97	1.12
PN13_3	Pinot noir	2013	4	0	195	72	872	9.23	1.35
PN13_4	Pinot noir	2013	5	7	202	79	968	11.86	1.62
PN13_5	Pinot noir	2013	6	14	209	86	1072	15.56	1.67
PN13_6	Pinot noir	2013	7	21	216	93	1156	17.69	1.82
PN13_7	Pinot noir	2013	8	28	223	100	1239	18.89	1.79
PN13_8	Pinot noir	2013	9	35	230	107	1346	22.04	1.96
PN13_9	Pinot noir	2013	10	42	237	114	1442	22.87	1.86
PN13_10	Pinot noir	2013	11	50	245	122	1564	28.84	1.66
CS13_0	Cabernet Sauvignon	2013	1	-26	177	47	517	0.48	0.59
CS13_1	Cabernet Sauvignon	2013	2	-20	183	53	629	0.44	0.67
CS13_2	Cabernet Sauvignon	2013	3	-13	190	60	743	0.48	0.76
CS13_3	Cabernet Sauvignon	2013	4	-6	197	67	834	2.00	0.76
CS13_4	Cabernet Sauvignon	2013	5	0	203	73	921	7.84	0.86
CS13_5	Cabernet Sauvignon	2013	6	8	211	81	1035	12.04	1.15
CS13_6	Cabernet Sauvignon	2013	7	15	218	88	1117	15.69	1.23
CS13_7	Cabernet Sauvignon	2013	8	22	225	95	1204	17.76	1.40
CS13_8	Cabernet Sauvignon	2013	9	29	232	102	1316	21.08	1.41
CS13_9	Cabernet Sauvignon	2013	10	36	239	109	1409	22.52	1.46
CS13_10	Cabernet Sauvignon	2013	11	43	246	116	1516	23.81	1.40
CS13_11	Cabernet Sauvignon	2013	12	49	252	122	1603	24.00	1.46
CS13_12	Cabernet Sauvignon	2013	13	56	259	129	1692	24.32	1.25
CS13_13	Cabernet Sauvignon	2013	14	63	266	136	1750	27.08	1.31
PN14_0	Pinot noir	2014	1	-27	167	50	502	0.52	0.80
PN14_1	Pinot noir	2014	2	-21	173	56	557	0.81	0.83
PN14_2	Pinot noir	2014	3	-14	180	63	648	1.34	0.96
PN14_3	Pinot noir	2014	4	-7	187	70	756	1.11	1.01
PN14_4	Pinot noir	2014	5	0	194	77	859	8.40	1.31
PN14_5	Pinot noir	2014	6	7	201	84	954	12.23	1.45
PN14_6	Pinot noir	2014	7	14	208	91	1056	14.44	1.72
PN14_7	Pinot noir	2014	8	21	215	98	1144	18.53	1.78
PN14_8	Pinot noir	2014	9	28	222	105	1239	19.85	1.79
PN14_9	Pinot noir	2014	10	35	229	112	1350	23.85	1.73
PN14_10	Pinot noir	2014	11	42	236	119	1448	25.71	1.89
PN14_11	Pinot noir	2014	12	45	239	122	1487	25.57	1.50
CS14_0	Cabernet Sauvignon	2014	1	-31	173	42	453	0.50	0.41
CS14_1	Cabernet Sauvignon	2014	2	-22	182	51	577	0.43	0.60
CS14_2	Cabernet Sauvignon	2014	3	-15	189	58	683	0.56	0.55
CS14_3	Cabernet Sauvignon	2014	4	-8	196	65	781	2.26	0.59
CS14_4	Cabernet Sauvignon	2014	5	0	204	73	896	7.68	0.76
CS14_5	Cabernet Sauvignon	2014	6	6	210	79	977	13.25	0.89
CS14_6	Cabernet Sauvignon	2014	7	13	217	86	1065	15.20	1.03
CS14_7	Cabernet Sauvignon	2014	8	20	224	93	1166	17.55	1.03
CS14_8	Cabernet Sauvignon	2014	9	27	231	100	1277	21.16	1.19
CS14_9	Cabernet Sauvignon	2014	10	34	238	107	1370	20.89	1.51
CS14_10	Cabernet Sauvignon	2014	11	41	245	114	1466	23.22	1.20
CS14_11	Cabernet Sauvignon	2014	12	48	252	121	1564	23.25	1.19
CS14_12	Cabernet Sauvignon	2014	13	55	259	128	1656	25.02	1.00

Table S2

Mapping of the PN and CS non-smoothed samples on the MPhS and relative values of MPhS stage variation by time.

Sample ID	MPhS stage	Δ MPhS / Δ time
PN12_0	3	–
PN12_1	5	0.250
PN12_2	4	-0.100
PN12_3	10	0.667
PN12_4	13	0.333
PN12_5	16	0.273
PN12_6	18	0.200
PN12_7	19	0.091
PN12_8	21	0.200
PN12_9	23	0.182
CS12_0	2	–
CS12_1	3	0.111
CS12_2	4	0.100
CS12_3	6	0.222
CS12_4	13	0.778
CS12_5	15	0.182
CS12_6	17	0.200
CS12_7	21	0.364
CS12_8	21	0.000
CS12_9	24	0.273
CS12_10	24	0.000
CS12_11	30	0.545
CS12_12	28	-0.333
PN13_0	6	–
PN13_1	6	0.000
PN13_2	9	0.429
PN13_3	14	0.714
PN13_4	16	0.286
PN13_5	17	0.143
PN13_6	18	0.143
PN13_7	20	0.286
PN13_8	21	0.143
PN13_9	23	0.286
PN13_10	25	0.250
CS13_0	3	–
CS13_1	4	0.167
CS13_2	5	0.143
CS13_3	10	0.714
CS13_4	13	0.500
CS13_5	16	0.375
CS13_6	18	0.286
CS13_7	20	0.286
CS13_8	21	0.143
CS13_9	23	0.286
CS13_10	24	0.143
CS13_11	25	0.167
CS13_12	26	0.143
CS13_13	29	0.429
PN14_0	1	–
PN14_1	4	0.500
PN14_2	7	0.429
PN14_3	12	0.714
PN14_4	14	0.286
PN14_5	16	0.286
PN14_6	17	0.143
PN14_7	18	0.143
PN14_8	19	0.143
PN14_9	22	0.429
PN14_10	24	0.286
PN14_11	23	-0.333
CS14_0	1	–
CS14_1	1	0.000
CS14_2	5	0.571
CS14_3	10	0.714
CS14_4	15	0.625
CS14_5	17	0.333
CS14_6	17	0.000
CS14_7	20	0.429
CS14_8	21	0.143
CS14_9	21	0.000
CS14_10	24	0.429
CS14_11	26	0.286
CS14_12	26	0.000

Table S3

Screening guidelines for the definition of the core set of genes.

Phase	Feature	Rule	Number of genes
I	Unexpressed genes	For each gene *i* and for each experimental condition *j*, let *g* be a function such that<br>$$g_{ji} = g(x_{j1i}, x_{j2i}, x_{j3i}) = \begin{cases} m_{ji}, & \text{if } \{x_{j1i} \geq 1 \vee x_{i2i} \geq 1 \vee x_{j3i} \geq 1\} \\ 0, & \text{otherwise} \end{cases}$$<br>We define<br>$$\nu_{hi} = \sum_{j \in J_h} I_1(g_{ji})$$<br>where $I_1(g_{ji})$ is the indicator function assuming value 0 if $g_{ji} \geq 1$ and 1 otherwise. Finally, let<br>$$\nu_i = \sum_{h=1}^{6} I_2(\nu_{hi})$$<br>where $I_2(\nu_{hi})$ is the indicator function assuming value 0 if $\nu_{hi} < H_h - 1$ and 1 otherwise.<br>Gene *i* is removed if $\nu_i \geq 1$.<br>Genes with $\nu_i < 1$ are checked with the same rule in *dataset B* and are removed if they are found to have $\nu_i \geq 1$.	12949
II	Genes not associated to the berry development	For each gene *i* and each *CxV* combination *h*, the variability of the expressions among the H_h timepoints was measured by means of the Pearson ratio<br>$$\eta_{hi}^2 = \frac{\sum_{j \in J_h} (m_{ji} - m_{hi})^2}{\sum_{r=1}^{3} \sum_{j \in J_h} (x_{jri} - m_{hi})^2}$$<br>We define $\eta_i^2 = min_h(\eta_{hi}^2)$.<br>Gene *i* is removed if $\eta_i^2 < 0.6$.<br>Genes with $\eta_i^2 \geq 0.6$ are checked with the same rule in *dataset B* and are removed if they are found to have $\eta_i^2 < 0.6$.	6893

Fig. S1

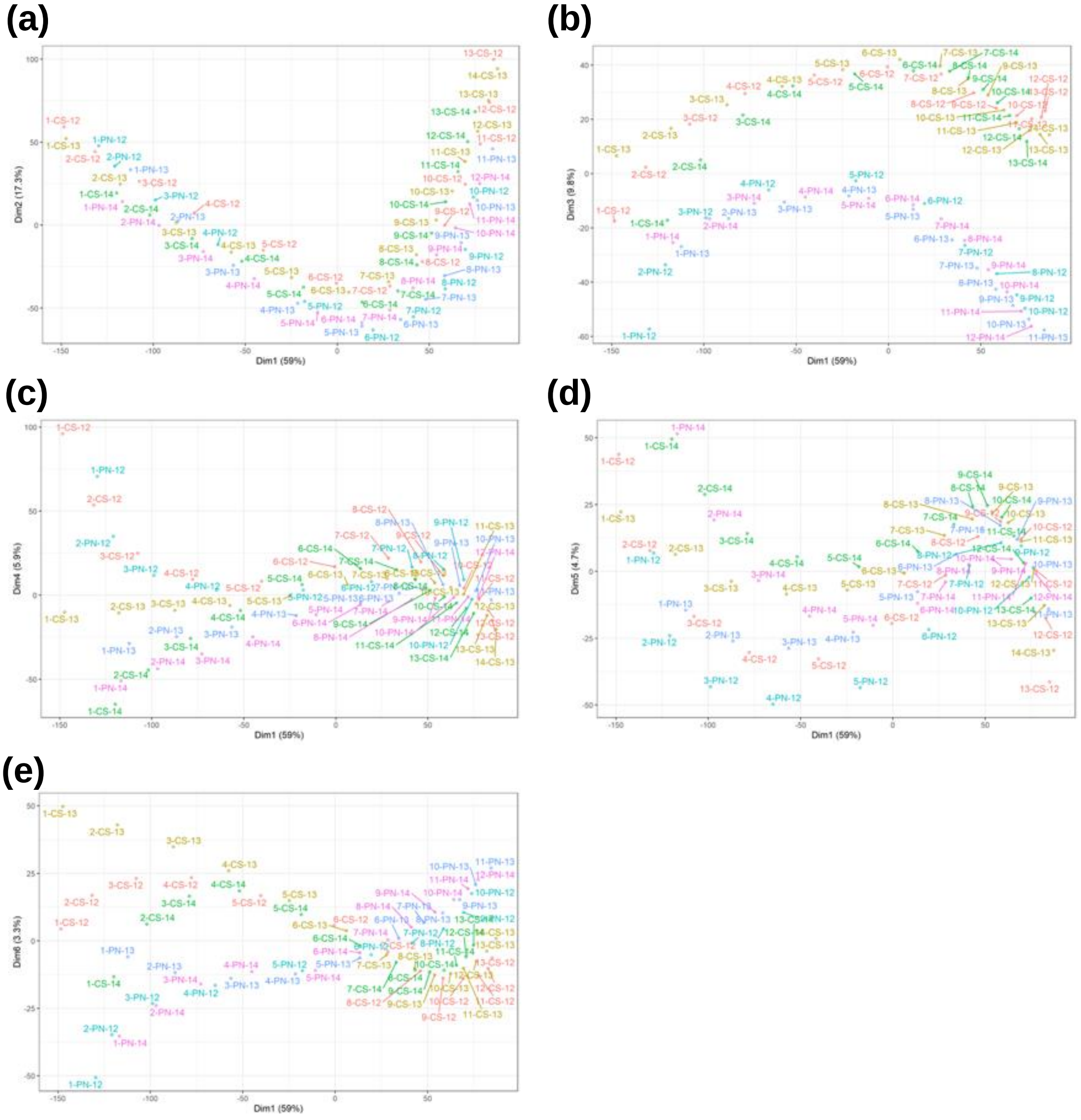

Samples distribution according to the six PCs. (a) PC1 (53.6%) by PC2 (15.8%). (b) PC1 by PC3 (9.8%). (c) PC1 by PC4 (5.9%). (d) PC1 by PC5 (4.7%). e. PC1 by PC6 (3.3%). The two cultivars exhibited different patterns by PC3 and PC6, whereas PC4 reveled separation between 2012 and the other vintages at the early time points. PC1, PC2 and PC5, with an overall 74% explained variance, accounted for different perspectives on the berry development, excluding cultivar and vintage effects.

Fig S2

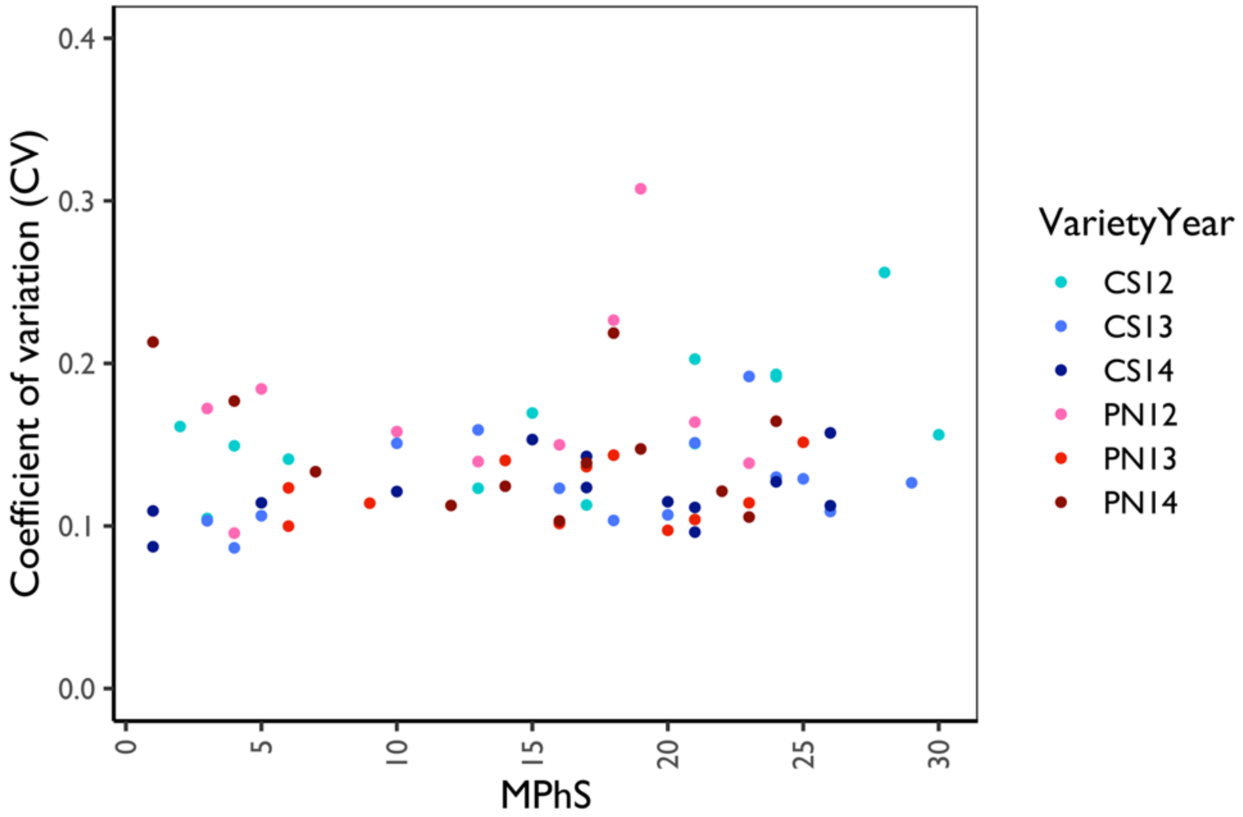

Average Coefficient of Variation values calculated for biological replicates of each sample of CS and PN time series, mapped on the MPhS.

Fig. S3

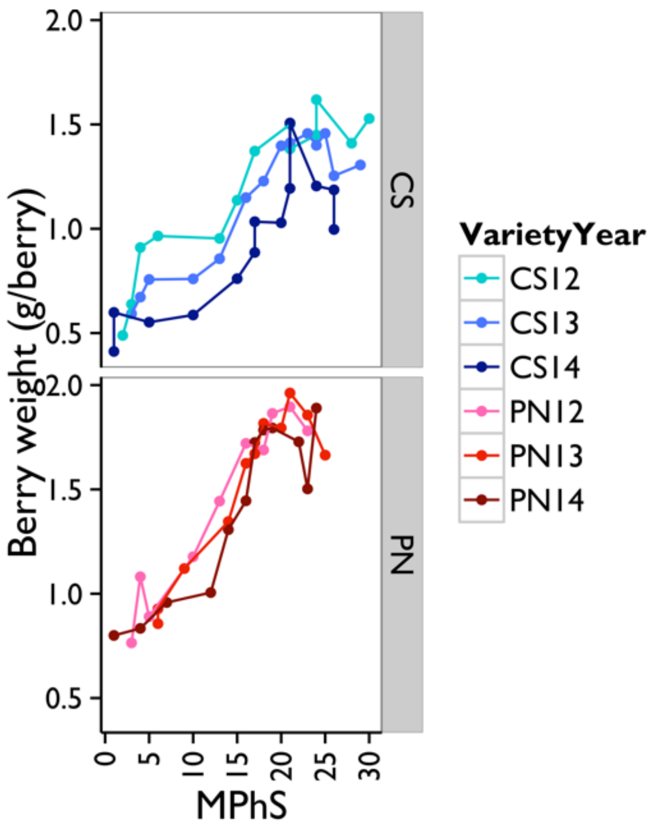

Trends of berry weight by MPhS, in CS and PN over the three years. Plots were generated using R package ggpplot2 version 2.2.

Fig. S4

(a)

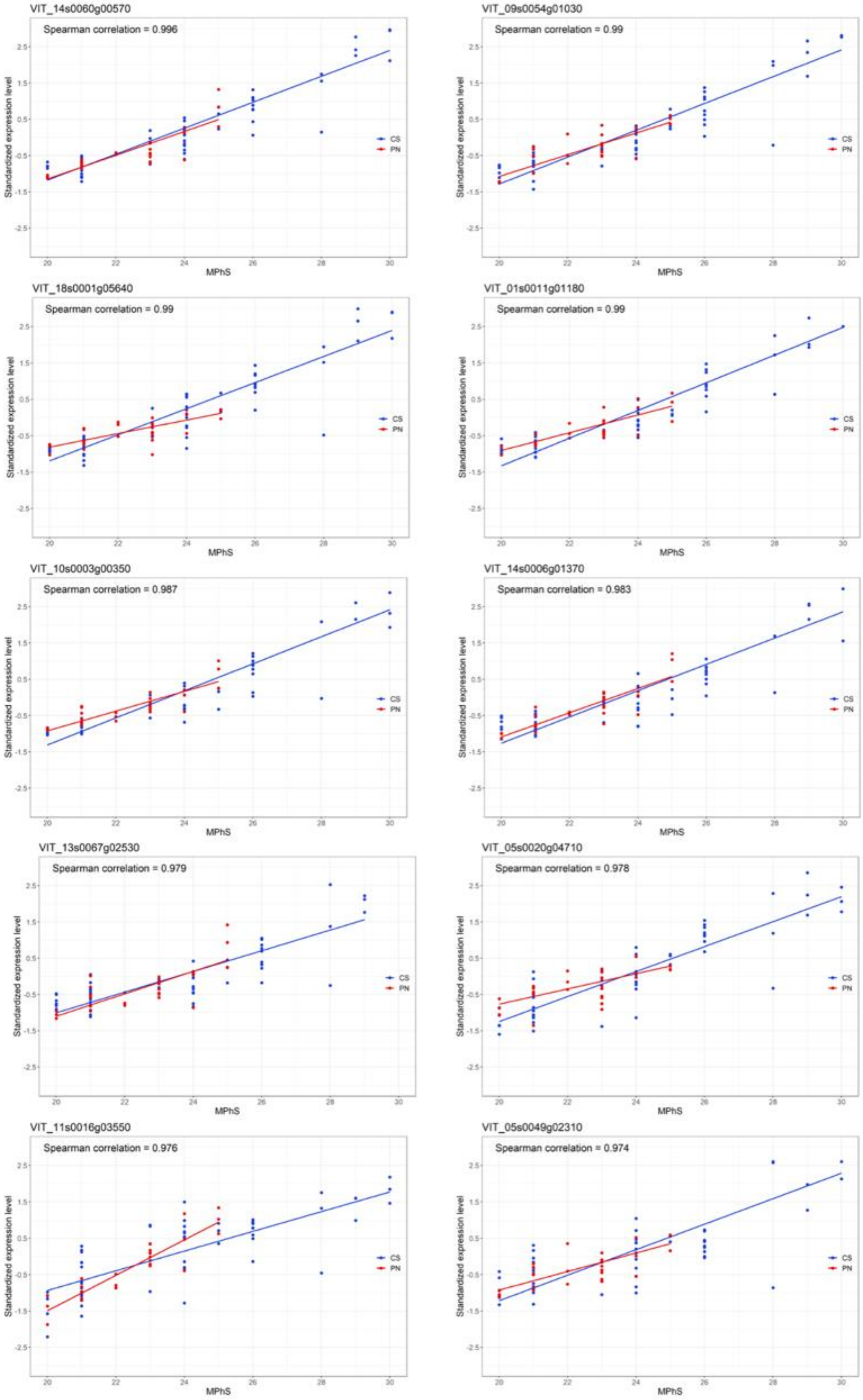
VIT_14s0060g00570
Spearman correlation = 0.996
VIT_09s0054g01030
Spearman correlation = 0.99
VIT_18s0001g05640
Spearman correlation = 0.99
VIT_01s0011g01180
Spearman correlation = 0.99
VIT_10s0003g00350
Spearman correlation = 0.987
VIT_14s0006g01370
Spearman correlation = 0.983
VIT_13s0067g02530
Spearman correlation = 0.979
VIT_05s0020g04710
Spearman correlation = 0.978
VIT_11s0016g03550
Spearman correlation = 0.976
VIT_05s0049g02310
Spearman correlation = 0.974
Standardized expression level
MPhS
CS
PN

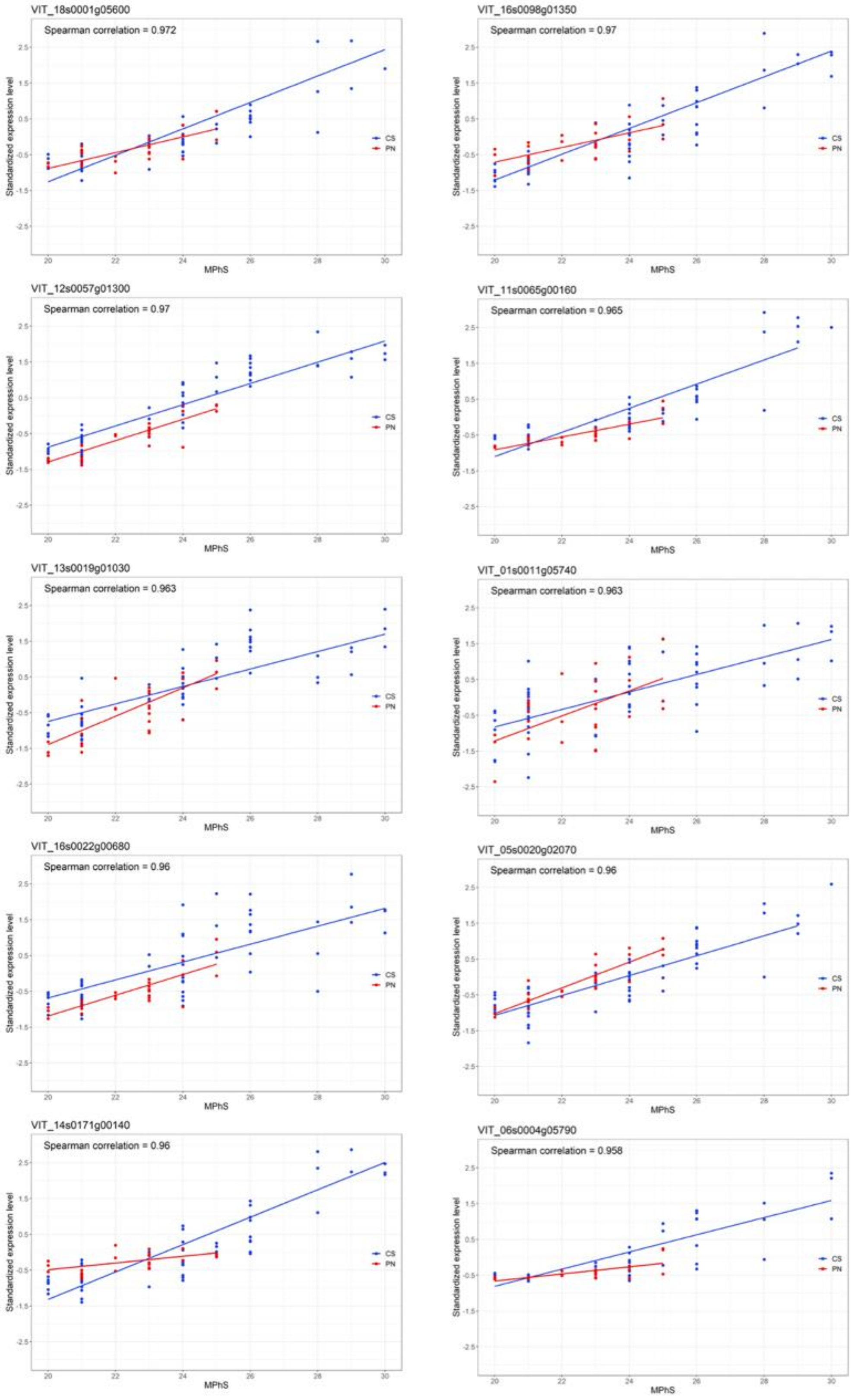
VIT_18s0001g05600
Spearman correlation = 0.972
VIT_16s0098g01350
Spearman correlation = 0.97
VIT_12s0057g01300
Spearman correlation = 0.97
VIT_11s0065g00160
Spearman correlation = 0.965
VIT_13s0019g01030
Spearman correlation = 0.963
VIT_01s0011g05740
Spearman correlation = 0.963
VIT_16s0022g00680
Spearman correlation = 0.96
VIT_05s0020g02070
Spearman correlation = 0.96
VIT_14s0171g00140
Spearman correlation = 0.96
VIT_06s0004g05790
Spearman correlation = 0.958
Standardized expression level
MPhS
CS
PN

(b)

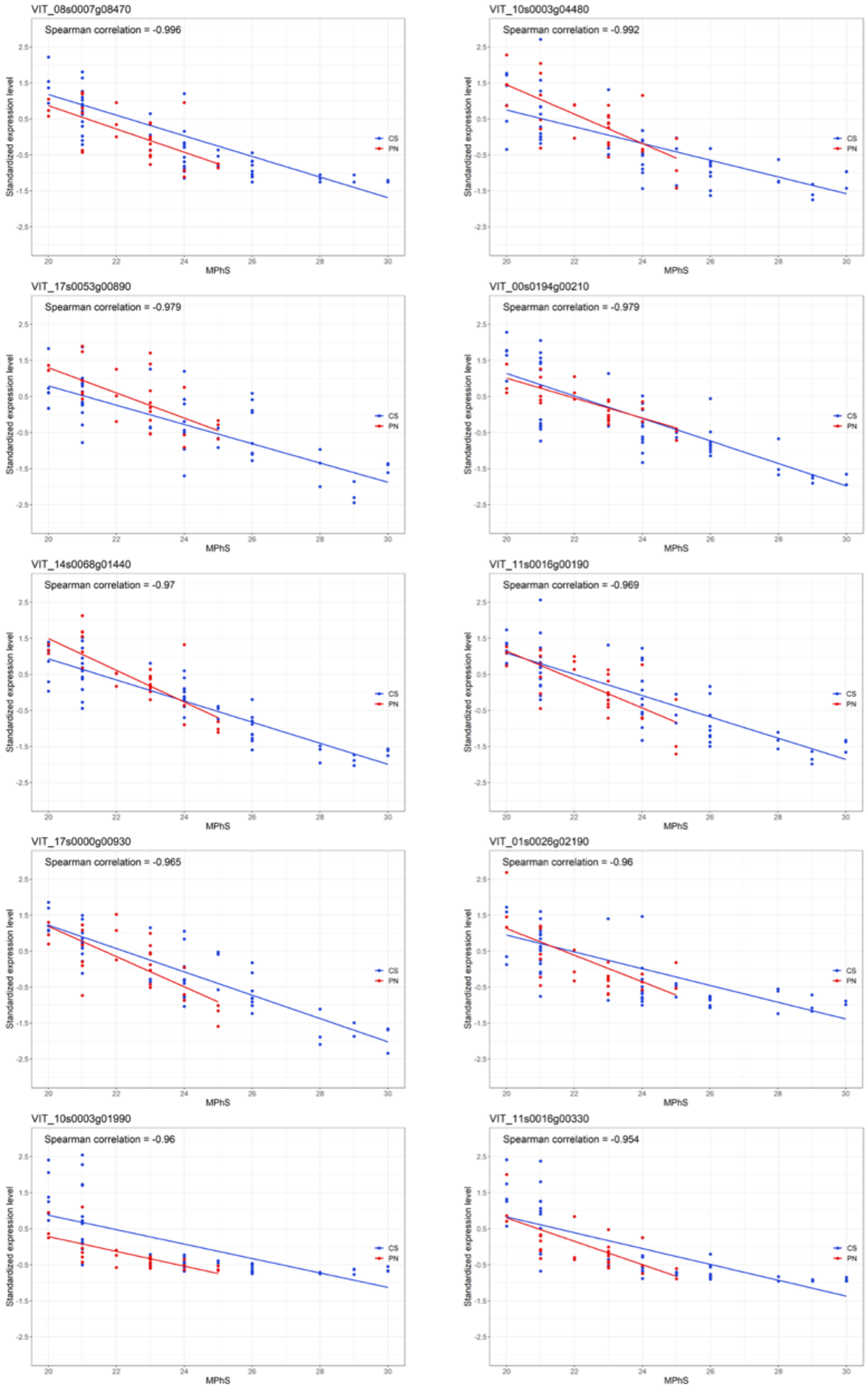
VIT_08s0007g08470
Spearman correlation = -0.996
VIT_10s0003g04480
Spearman correlation = -0.992
VIT_17s0053g00890
Spearman correlation = -0.979
VIT_00s0194g00210
Spearman correlation = -0.979
VIT_14s0068g01440
Spearman correlation = -0.97
VIT_11s0016g00190
Spearman correlation = -0.969
VIT_17s0000g00930
Spearman correlation = -0.965
VIT_01s0026g02190
Spearman correlation = -0.96
VIT_10s0003g01990
Spearman correlation = -0.96
VIT_11s0016g00330
Spearman correlation = -0.954
Standardized expression level
MPhS
CS
PN

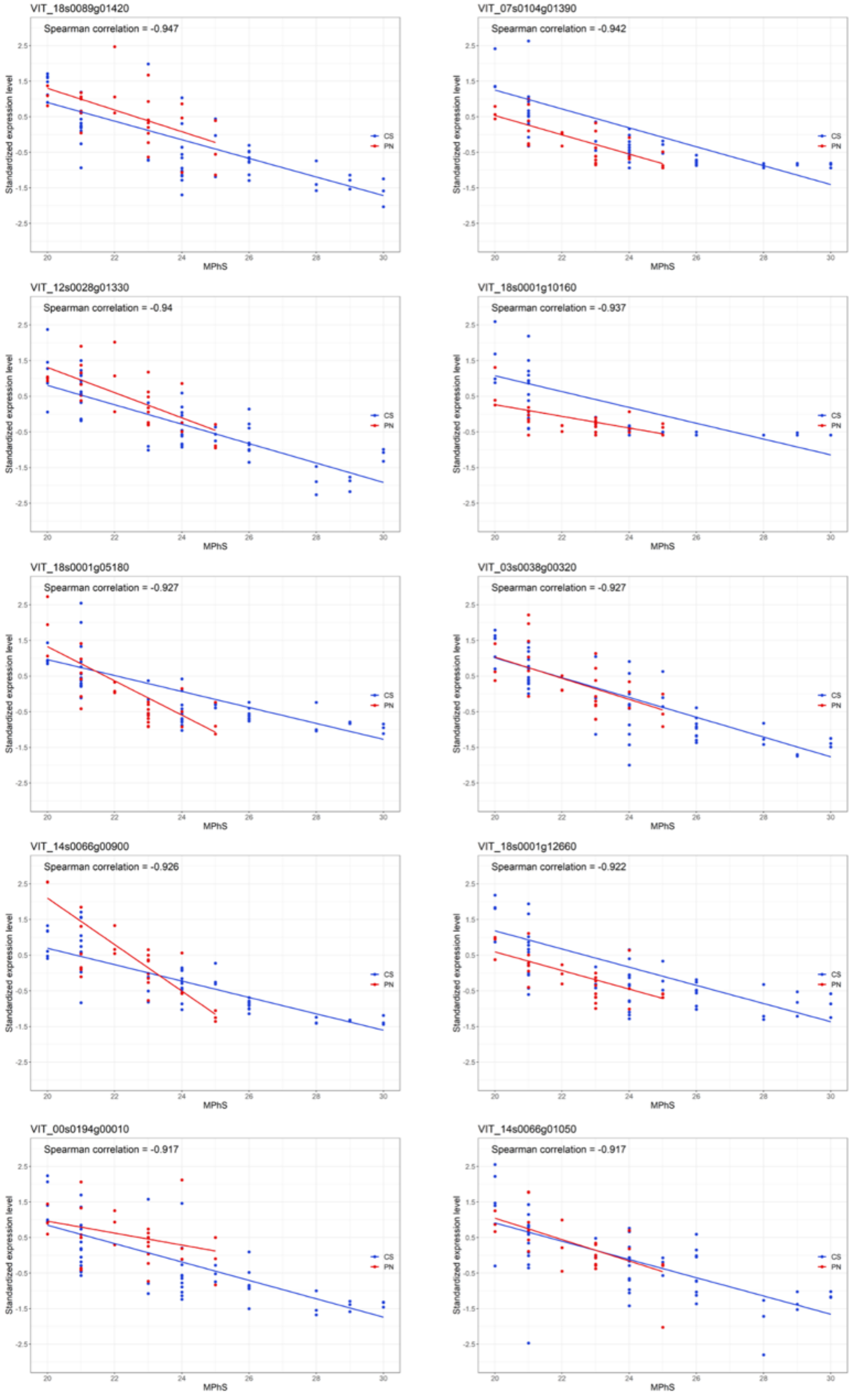

Trends of expression values of the top-20 most positively correlated (a) and the top-20 most negatively correlated (b) genes with the 20-to-30 MPhS stage progression in CS and PN. Solid lines depict the regression line. The estimated Spearman's rank correlation coefficient is also reported.

Fig. S5

(a)

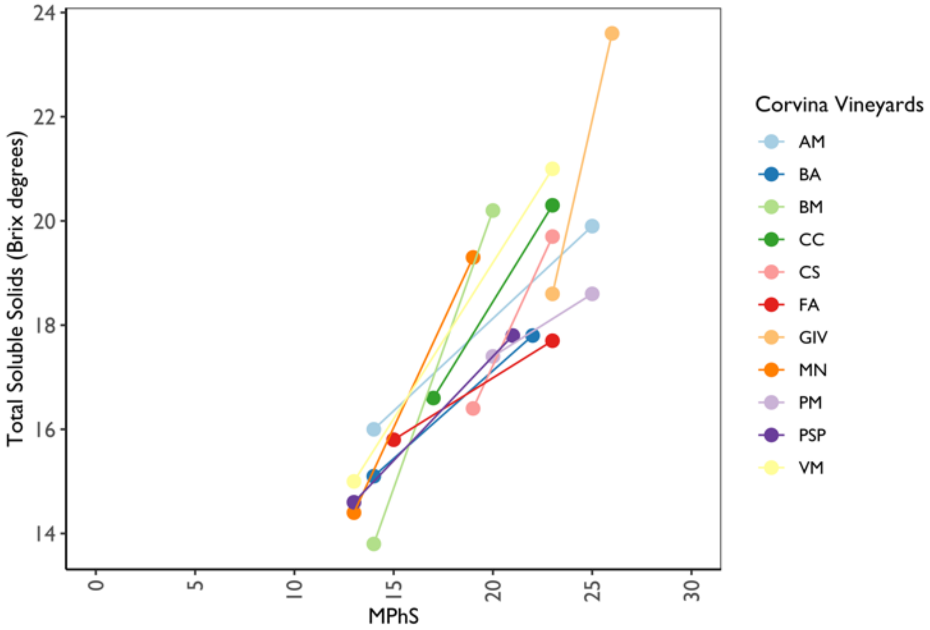

(b)

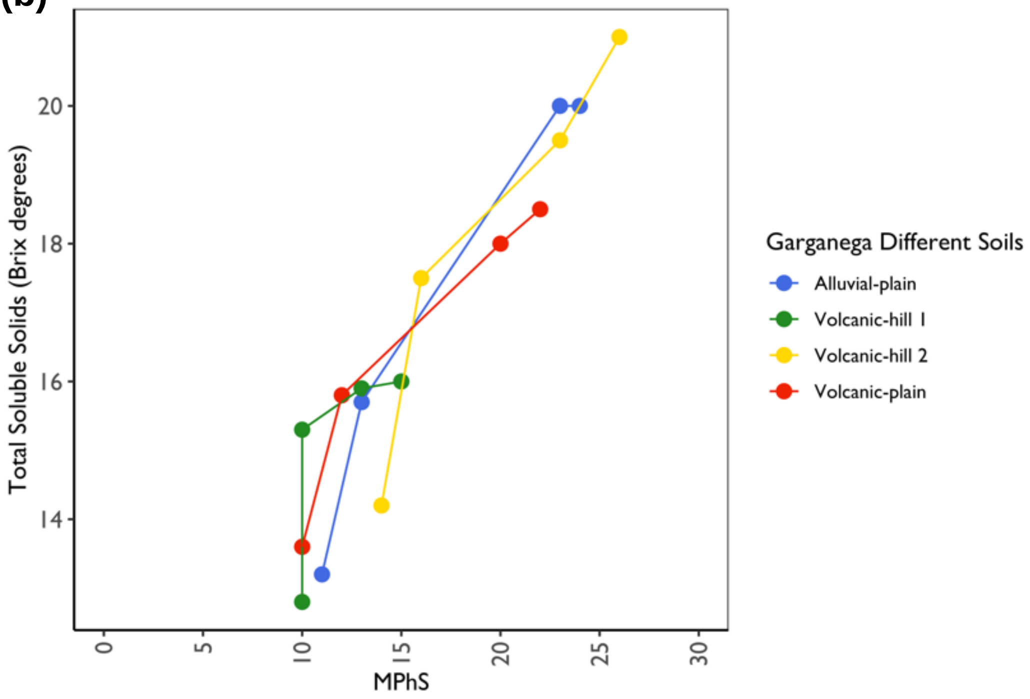

Trends of percentage of reducing sugars accumulation by MPhS in (a) cv Corvina (Dal Santo et al., 2013) and in (b) cv Garganega (Dal Santo et al., 2016).

Fig. S6

(a)

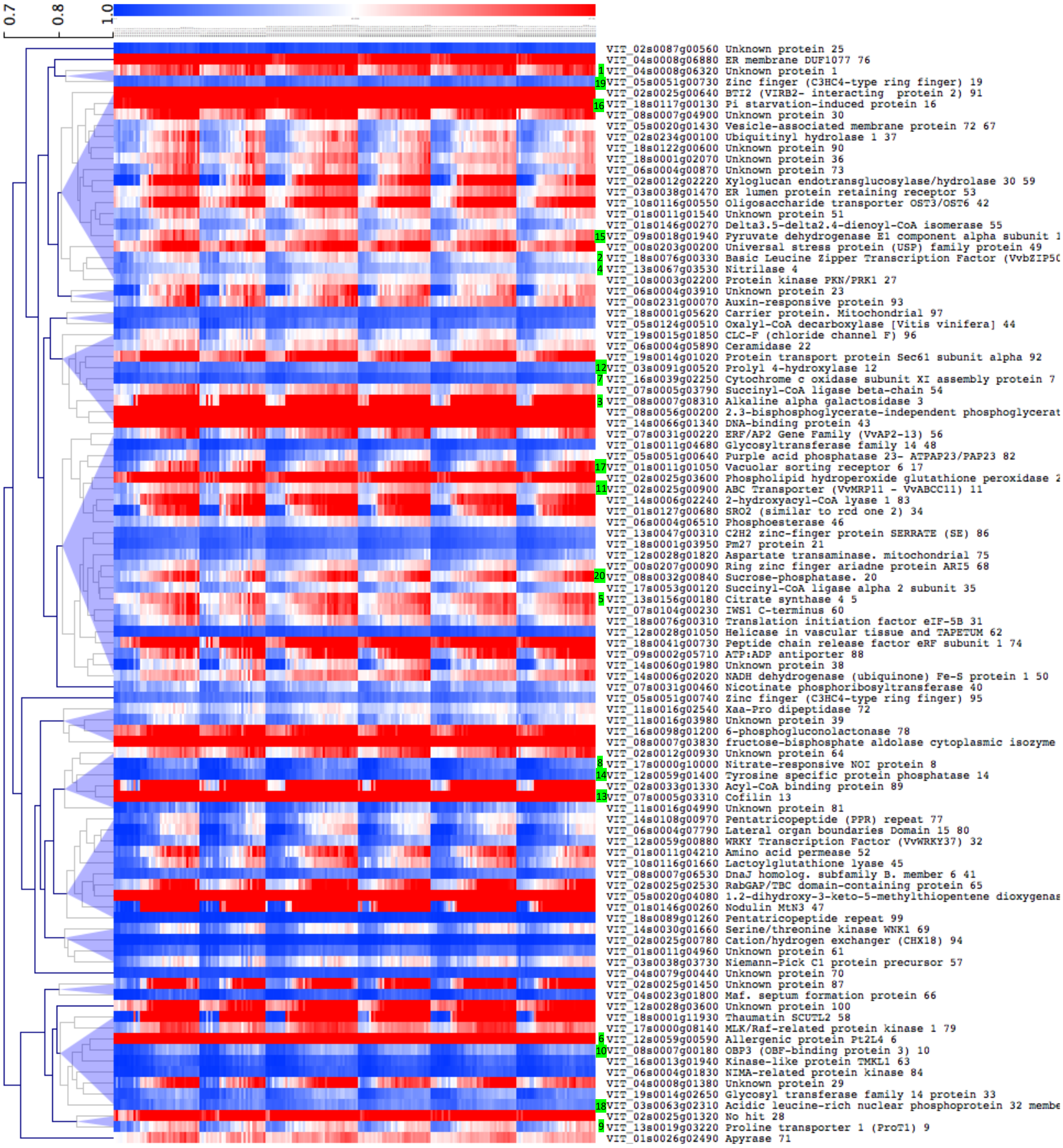
0.7
0.8
1.0
VIT_02s0087g00560 Unknown protein 25
VIT_04s0008g06880 ER membrane DUF1077 76
1 VIT_04s0008g06320 Unknown protein 1
19 VIT_05s0051g00730 Zinc finger (C3HC4-type ring finger) 19
VIT_02s0025g00640 BTI2 (VIRB2- interacting protein 2) 91
16 VIT_18s0117g00130 Pi starvation-induced protein 16
VIT_08s0007g04900 Unknown protein 30
VIT_05s0020g01430 Vesicle-associated membrane protein 72 67
VIT_02s0234g00100 Ubiquitinyl hydrolase 1 37
VIT_18s0122g00600 Unknown protein 90
VIT_18s0001g02070 Unknown protein 36
VIT_06s0004g00870 Unknown protein 73
VIT_02s0012g02220 Xyloglucan endotransglucosylase/hydrolase 30 59
VIT_03s0038g01470 ER lumen protein retaining receptor 53
VIT_10s0116g00550 Oligosaccharide transporter OST3/OST6 42
VIT_01s0011g01540 Unknown protein 51
VIT_01s0146g00270 Delta3.5-delta2.4-dienoyl-CoA isomerase 55
15 VIT_09s0018g01940 Pyruvate dehydrogenase E1 component alpha subunit 1
VIT_00s0203g00200 Universal stress protein (USP) family protein 49
2 VIT_18s0076g00330 Basic Leucine Zipper Transcription Factor (VvbZIP50
4 VIT_13s0067g03530 Nitrilase 4
VIT_10s0003g02200 Protein kinase PKN/PRK1 27
VIT_06s0004g03910 Unknown protein 23
VIT_00s0231g00070 Auxin-responsive protein 93
VIT_18s0001g05620 Carrier protein. Mitochondrial 97
VIT_05s0124g00510 Oxalyl-CoA decarboxylase [Vitis vinifera] 44
VIT_19s0015g01850 CLC-F (chloride channel F) 96
VIT_06s0004g05890 Ceramidase 22
VIT_19s0014g01020 Protein transport protein Sec61 subunit alpha 92
12 VIT_03s0091g00520 Prolyl 4-hydroxylase 12
7 VIT_16s0039g02250 Cytochrome c oxidase subunit XI assembly protein 7
VIT_07s0005g03790 Succinyl-CoA ligase beta-chain 54
3 VIT_08s0007g08310 Alkaline alpha galactosidase 3
VIT_08s0056g00200 2.3-bisphosphoglycerate-independent phosphoglycerat
VIT_14s0066g01340 DNA-binding protein 43
VIT_07s0031g00220 ERF/AP2 Gene Family (VvAP2-13) 56
VIT_01s0011g04680 Glycosyltransferase family 14 48
VIT_05s0051g00640 Purple acid phosphatase 23- ATPAP23/PAP23 82
17 VIT_01s0011g01050 Vacuolar sorting receptor 6 17
VIT_02s0025g03600 Phospholipid hydroperoxide glutathione peroxidase 2
11 VIT_02s0025g00900 ABC Transporter (VvMRP11 - VvABCC11) 11
VIT_14s0006g02240 2-hydroxyacyl-CoA lyase 1 83
VIT_01s0127g00680 SRO2 (similar to rcd one 2) 34
VIT_06s0004g06510 Phosphoesterase 46
VIT_13s0047g00310 C2H2 zinc-finger protein SERRATE (SE) 86
VIT_18s0001g03950 Pm27 protein 21
VIT_12s0028g01820 Aspartate transaminase. mitochondrial 75
VIT_00s0207g00090 Ring zinc finger ariadne protein ARI5 68
20 VIT_08s0032g00840 Sucrose-phosphatase. 20
VIT_17s0053g00120 Succinyl-CoA ligase alpha 2 subunit 35
5 VIT_13s0156g00180 Citrate synthase 4 5
VIT_07s0104g00230 IWS1 C-terminus 60
VIT_18s0076g00310 Translation initiation factor eIF-5B 31
VIT_12s0028g01050 Helicase in vascular tissue and TAPETUM 62
VIT_18s0041g00730 Peptide chain release factor eRF subunit 1 74
VIT_09s0002g05710 ATP:ADP antiporter 88
VIT_14s0060g01980 Unknown protein 38
VIT_14s0006g02020 NADH dehydrogenase (ubiquinone) Fe-S protein 1 50
VIT_07s0031g00460 Nicotinate phosphoribosyltransferase 40
VIT_05s0051g00740 Zinc finger (C3HC4-type ring finger) 95
VIT_11s0016g02540 Xaa-Pro dipeptidase 72
VIT_11s0016g03980 Unknown protein 39
VIT_16s0098g01200 6-phosphogluconolactonase 78
VIT_08s0007g03830 fructose-bisphosphate aldolase cytoplasmic isozyme
VIT_02s0012g00930 Unknown protein 64
8 VIT_17s0000g10000 Nitrate-responsive NOI protein 8
14 VIT_12s0059g01400 Tyrosine specific protein phosphatase 14
VIT_02s0033g01330 Acyl-CoA binding protein 89
13 VIT_07s0005g03310 Cofilin 13
VIT_11s0016g04990 Unknown protein 81
VIT_14s0108g00970 Pentatricopeptide (PPR) repeat 77
VIT_06s0004g07790 Lateral organ boundaries Domain 15 80
VIT_12s0059g00880 WRKY Transcription Factor (VvWRKY37) 32
VIT_01s0011g04210 Amino acid permease 52
VIT_10s0116g01660 Lactoylglutathione lyase 45
VIT_08s0007g06530 DnaJ homolog. subfamily B. member 6 41
VIT_02s0025g02530 RabGAP/TBC domain-containing protein 65
VIT_05s0020g04080 1.2-dihydroxy-3-keto-5-methylthiopentene dioxygenas
VIT_01s0146g00260 Nodulin MtN3 47
VIT_18s0089g01260 Pentatricopeptide repeat 99
VIT_14s0030g01660 Serine/threonine kinase WNK1 69
VIT_02s0025g00780 Cation/hydrogen exchanger (CHX18) 94
VIT_01s0011g04960 Unknown protein 61
VIT_03s0038g03730 Niemann-Pick C1 protein precursor 57
VIT_04s0079g00440 Unknown protein 70
VIT_02s0025g01450 Unknown protein 87
VIT_04s0023g01800 Maf. septum formation protein 66
VIT_12s0028g03600 Unknown protein 100
VIT_18s0001g11930 Thaumatin SCUTL2 58
VIT_17s0000g08140 MLK/Raf-related protein kinase 1 79
6 VIT_12s0059g00590 Allergenic protein Pt2L4 6
10 VIT_08s0007g00180 OBP3 (OBF-binding protein 3) 10
VIT_16s0013g01940 Kinase-like protein TMKL1 63
VIT_06s0004g01830 NIMA-related protein kinase 84
VIT_04s0008g01380 Unknown protein 29
VIT_19s0014g02650 Glycosyl transferase family 14 protein 33
18 VIT_03s0063g02310 Acidic leucine-rich nuclear phosphoprotein 32 membe
VIT_02s0025g01320 No hit 28
9 VIT_13s0019g03220 Proline transporter 1 (ProT1) 9
VIT_01s0026g02490 Apyrase 71

(b)

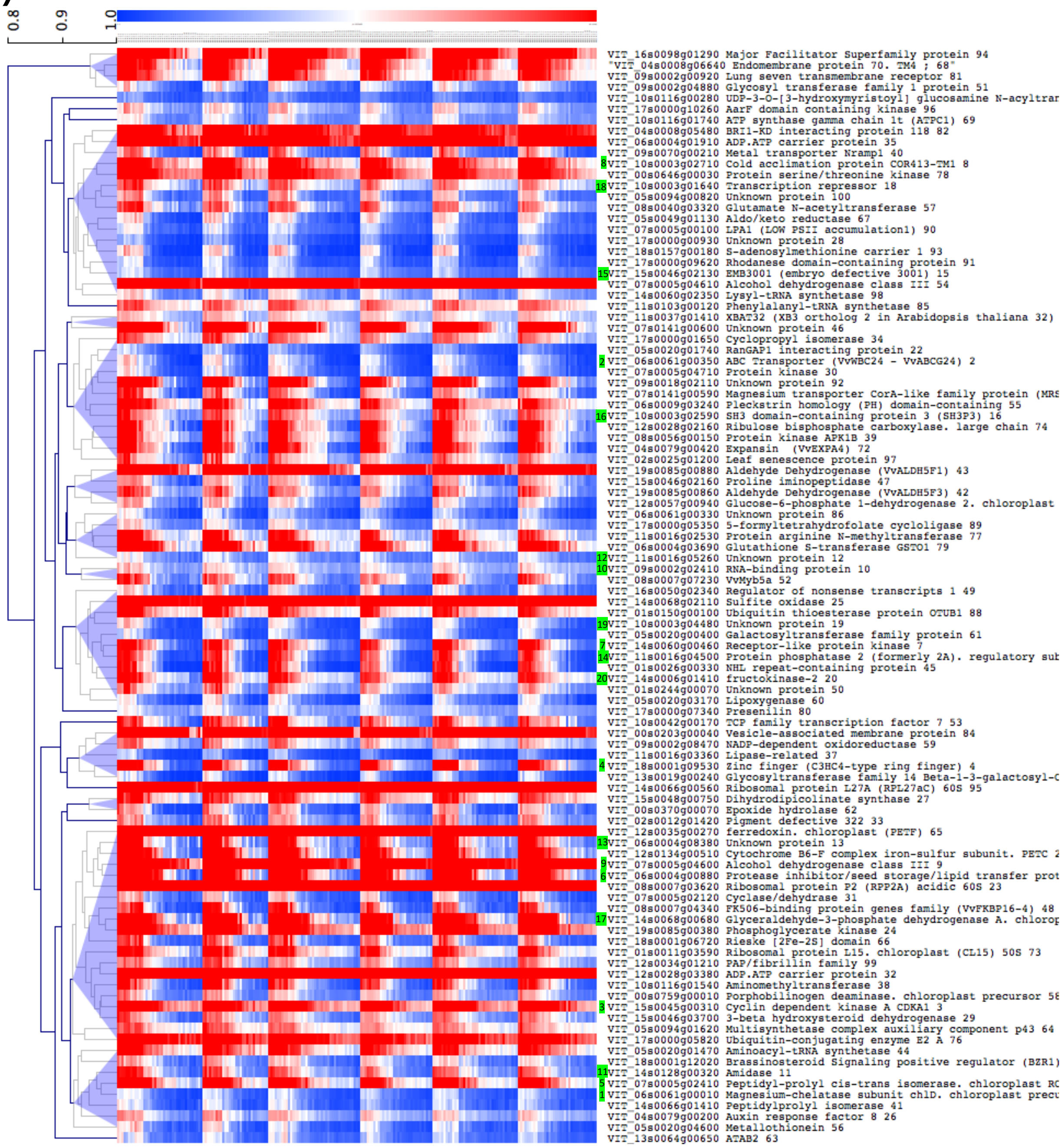

0.8
0.9
1.0
VIT_16s0098g01290 Major Facilitator Superfamily protein 94
"VIT_04s0008g06640 Endomembrane protein 70. TM4 ; 68"
VIT_09s0002g00920 Lung seven transmembrane receptor 81
VIT_09s0002g04880 Glycosyl transferase family 1 protein 51
VIT_10s0116g00280 UDP-3-O-[3-hydroxymyristoyl] glucosamine N-acyltra
VIT_17s0000g10260 AarF domain containing kinase 96
VIT_10s0116g01740 ATP synthase gamma chain 1t (ATPC1) 69
VIT_04s0008g05480 BRI1-KD interacting protein 118 82
VIT_06s0004g01910 ADP.ATP carrier protein 35
VIT_09s0070g00210 Metal transporter Nramp1 40
8 VIT_10s0003g02710 Cold acclimation protein COR413-TM1 8
VIT_00s0646g00030 Protein serine/threonine kinase 78
18 VIT_10s0003g01640 Transcription repressor 18
VIT_05s0094g00820 Unknown protein 100
VIT_08s0040g03320 Glutamate N-acetyltransferase 57
VIT_05s0049g01130 Aldo/keto reductase 67
VIT_07s0005g00100 LPA1 (LOW PSII accumulation1) 90
VIT_17s0000g00930 Unknown protein 28
VIT_18s0157g00180 S-adenosylmethionine carrier 1 93
VIT_17s0000g09620 Rhodanese domain-containing protein 91
15 VIT_15s0046g02130 EMB3001 (embryo defective 3001) 15
VIT_07s0005g04610 Alcohol dehydrogenase class III 54
VIT_14s0060g02350 Lysyl-tRNA synthetase 98
VIT_11s0103g00120 Phenylalanyl-tRNA synthetase 85
VIT_11s0037g01410 XBAT32 (XB3 ortholog 2 in Arabidopsis thaliana 32)
VIT_07s0141g00600 Unknown protein 46
VIT_17s0000g01650 Cyclopropyl isomerase 34
VIT_05s0020g01740 RanGAP1 interacting protein 22
2 VIT_06s0061g00350 ABC Transporter (VvWBC24 - VvABCG24) 2
VIT_07s0005g04710 Protein kinase 30
VIT_09s0018g02110 Unknown protein 92
VIT_07s0141g00590 Magnesium transporter CorA-like family protein (MR
VIT_06s0009g03240 Pleckstrin homology (PH) domain-containing 55
16 VIT_10s0003g02590 SH3 domain-containing protein 3 (SH3P3) 16
VIT_12s0028g02160 Ribulose bisphosphate carboxylase. large chain 74
VIT_08s0056g00150 Protein kinase APK1B 39
VIT_04s0079g00420 Expansin (VvEXPA4) 72
VIT_02s0025g01200 Leaf senescence protein 97
VIT_19s0085g00880 Aldehyde Dehydrogenase (VvALDH5F1) 43
VIT_15s0046g02160 Proline iminopeptidase 47
VIT_19s0085g00860 Aldehyde Dehydrogenase (VvALDH5F3) 42
VIT_12s0057g00940 Glucose-6-phosphate 1-dehydrogenase 2. chloroplast
VIT_06s0061g00330 Unknown protein 86
VIT_17s0000g05350 5-formyltetrahydrofolate cycloligase 89
VIT_11s0016g02530 Protein arginine N-methyltransferase 77
VIT_06s0004g03690 Glutathione S-transferase GSTO1 79
12 VIT_11s0016g05260 Unknown protein 12
10 VIT_09s0002g02410 RNA-binding protein 10
VIT_08s0007g07230 VvMyb5a 52
VIT_16s0050g02340 Regulator of nonsense transcripts 1 49
VIT_14s0068g02110 Sulfite oxidase 25
VIT_01s0150g00100 Ubiquitin thioesterase protein OTUB1 88
19 VIT_10s0003g04480 Unknown protein 19
VIT_05s0020g00400 Galactosyltransferase family protein 61
7 VIT_14s0060g00460 Receptor-like protein kinase 7
14 VIT_11s0016g04500 Protein phosphatase 2 (formerly 2A). regulatory su
VIT_01s0026g00330 NHL repeat-containing protein 45
20 VIT_14s0006g01410 fructokinase-2 20
VIT_01s0244g00070 Unknown protein 50
VIT_05s0020g03170 Lipoxygenase 60
VIT_17s0000g07340 Presenilin 80
VIT_10s0042g00170 TCP family transcription factor 7 53
VIT_00s0203g00040 Vesicle-associated membrane protein 84
VIT_09s0002g08470 NADP-dependent oxidoreductase 59
VIT_11s0016g03360 Lipase-related 37
4 VIT_18s0001g09530 Zinc finger (C3HC4-type ring finger) 4
VIT_13s0019g00240 Glycosyltransferase family 14 Beta-1-3-galactosyl-
VIT_14s0066g00560 Ribosomal protein L27A (RPL27aC) 60S 95
VIT_15s0048g00750 Dihydrodipicolinate synthase 27
VIT_00s0370g00070 Epoxide hydrolase 62
VIT_02s0012g01420 Pigment defective 322 33
VIT_12s0035g00270 ferredoxin. chloroplast (PETF) 65
13 VIT_06s0004g08380 Unknown protein 13
VIT_12s0134g00510 Cytochrome B6-F complex iron-sulfur subunit. PETC
9 VIT_07s0005g04600 Alcohol dehydrogenase class III 9
6 VIT_06s0004g00880 Protease inhibitor/seed storage/lipid transfer prot
VIT_08s0007g03620 Ribosomal protein P2 (RPP2A) acidic 60S 23
VIT_07s0005g02120 Cyclase/dehydrase 31
VIT_08s0007g04340 FK506-binding protein genes family (VvFKBP16-4) 48
17 VIT_14s0068g00680 Glyceraldehyde-3-phosphate dehydrogenase A. chloro
VIT_19s0085g00380 Phosphoglycerate kinase 24
VIT_18s0001g06720 Rieske [2Fe-2S] domain 66
VIT_01s0011g03590 Ribosomal protein L15. chloroplast (CL15) 50S 73
VIT_12s0034g01210 PAP/fibrillin family 99
VIT_12s0028g03380 ADP.ATP carrier protein 32
VIT_10s0116g01540 Aminomethyltransferase 38
VIT_00s0759g00010 Porphobilinogen deaminase. chloroplast precursor 5
3 VIT_15s0045g00310 Cyclin dependent kinase A CDKA1 3
VIT_15s0046g03700 3-beta hydroxysteroid dehydrogenase 29
VIT_05s0094g01620 Multisynthetase complex auxiliary component p43 64
VIT_17s0000g05820 Ubiquitin-conjugating enzyme E2 A 76
VIT_05s0020g01470 Aminoacyl-tRNA synthetase 44
VIT_18s0001g12020 Brassinosteroid Signaling positive regulator (BZR1)
11 VIT_14s0128g00320 Amidase 11
5 VIT_07s0005g02410 Peptidyl-prolyl cis-trans isomerase. chloroplast R
1 VIT_06s0061g00010 Magnesium-chelatase subunit chlD. chloroplast prec
VIT_14s0066g01410 Peptidylprolyl isomerase 41
VIT_04s0079g00200 Auxin response factor 8 26
VIT_05s0020g04600 Metallothionein 56
VIT_13s0064g00650 ATAB2 63

(c)

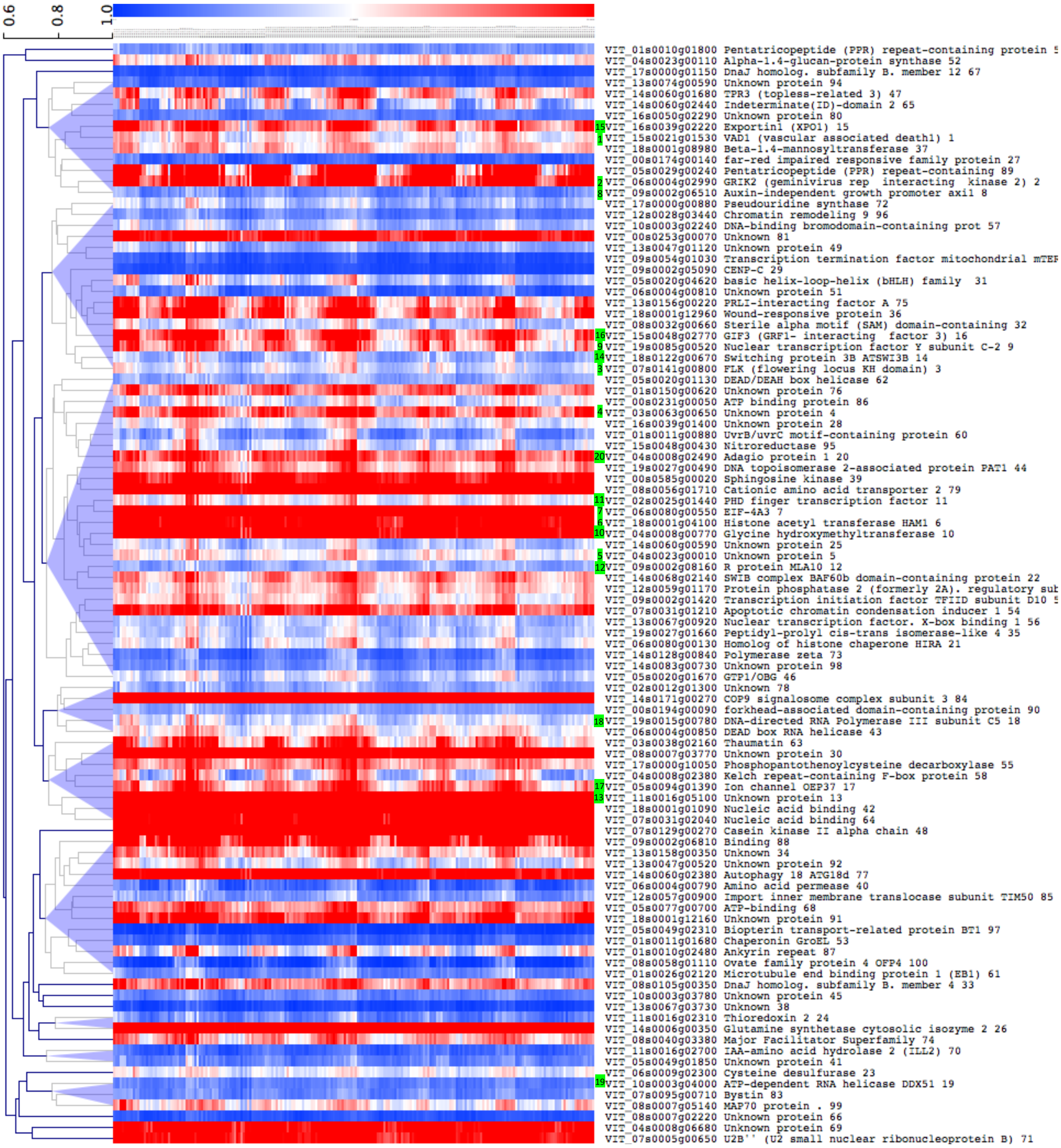

0.6
0.8
1.0
VIT_01s0010g01800 Pentatricopeptide (PPR) repeat-containing protein
VIT_04s0023g00110 Alpha-1.4-glucan-protein synthase 52
VIT_17s0000g01150 DnaJ homolog. subfamily B. member 12 67
VIT_13s0074g00590 Unknown protein 94
VIT_14s0060g01680 TPR3 (topless-related 3) 47
VIT_14s0060g02440 Indeterminate(ID)-domain 2 65
VIT_16s0050g02290 Unknown protein 80
15 VIT_16s0039g02220 Exportin1 (XPO1) 15
1 VIT_15s0021g01530 VAD1 (vascular associated death1) 1
VIT_18s0001g08980 Beta-1.4-mannosyltransferase 37
VIT_00s0174g00140 far-red impaired responsive family protein 27
VIT_05s0029g00240 Pentatricopeptide (PPR) repeat-containing 89
2 VIT_06s0004g02990 GRIK2 (geminivirus rep interacting kinase 2) 2
8 VIT_09s0002g06510 Auxin-independent growth promoter axi1 8
VIT_17s0000g00880 Pseudouridine synthase 72
VIT_12s0028g03440 Chromatin remodeling 9 96
VIT_10s0003g02240 DNA-binding bromodomain-containing prot 57
VIT_00s0253g00070 Unknown 81
VIT_13s0047g01120 Unknown protein 49
VIT_09s0054g01030 Transcription termination factor mitochondrial mTEF
VIT_09s0002g05090 CENP-C 29
VIT_05s0020g04620 basic helix-loop-helix (bHLH) family 31
VIT_06s0004g00810 Unknown protein 51
VIT_13s0156g00220 PRLI-interacting factor A 75
VIT_18s0001g12960 Wound-responsive protein 36
VIT_08s0032g00660 Sterile alpha motif (SAM) domain-containing 32
16 VIT_15s0048g02770 GIF3 (GRF1- interacting factor 3) 16
9 VIT_19s0085g00520 Nuclear transcription factor Y subunit C-2 9
14 VIT_18s0122g00670 Switching protein 3B ATSWI3B 14
3 VIT_07s0141g00800 FLK (flowering locus KH domain) 3
VIT_05s0020g01130 DEAD/DEAH box helicase 62
VIT_01s0150g00620 Unknown protein 76
VIT_00s0231g00050 ATP binding protein 86
4 VIT_03s0063g00650 Unknown protein 4
VIT_16s0039g01400 Unknown protein 28
VIT_01s0011g00880 UvrB/uvrC motif-containing protein 60
VIT_15s0048g00430 Nitroreductase 95
20 VIT_04s0008g02490 Adagio protein 1 20
VIT_19s0027g00490 DNA topoisomerase 2-associated protein PAT1 44
VIT_00s0585g00020 Sphingosine kinase 39
VIT_08s0056g01710 Cationic amino acid transporter 2 79
11 VIT_02s0025g01440 PHD finger transcription factor 11
7 VIT_06s0080g00550 EIF-4A3 7
6 VIT_18s0001g04100 Histone acetyl transferase HAM1 6
10 VIT_04s0008g00770 Glycine hydroxymethyltransferase 10
VIT_14s0060g00590 Unknown protein 25
5 VIT_04s0023g00010 Unknown protein 5
12 VIT_09s0002g08160 R protein MLA10 12
VIT_14s0068g02140 SWIB complex BAF60b domain-containing protein 22
VIT_12s0059g01170 Protein phosphatase 2 (formerly 2A). regulatory sub
VIT_09s0002g01420 Transcription initiation factor TFIID subunit D10
VIT_07s0031g01210 Apoptotic chromatin condensation inducer 1 54
VIT_13s0067g00920 Nuclear transcription factor. X-box binding 1 56
VIT_19s0027g01660 Peptidyl-prolyl cis-trans isomerase-like 4 35
VIT_06s0080g00130 Homolog of histone chaperone HIRA 21
VIT_14s0128g00840 Polymerase zeta 73
VIT_14s0083g00730 Unknown protein 98
VIT_05s0020g01670 GTP1/OBG 46
VIT_02s0012g01300 Unknown 78
VIT_14s0171g00270 COP9 signalosome complex subunit 3 84
VIT_00s0194g00090 forkhead-associated domain-containing protein 90
18 VIT_19s0015g00780 DNA-directed RNA Polymerase III subunit C5 18
VIT_06s0004g00850 DEAD box RNA helicase 43
VIT_03s0038g02160 Thaumatin 63
VIT_08s0007g03770 Unknown protein 30
VIT_17s0000g10050 Phosphopantothenoylcysteine decarboxylase 55
VIT_04s0008g02380 Kelch repeat-containing F-box protein 58
17 VIT_05s0094g01390 Ion channel OEP37 17
13 VIT_11s0016g05100 Unknown protein 13
VIT_18s0001g01090 Nucleic acid binding 42
VIT_07s0031g02040 Nucleic acid binding 64
VIT_07s0129g00270 Casein kinase II alpha chain 48
VIT_09s0002g06810 Binding 88
VIT_13s0158g00350 Unknown 34
VIT_13s0047g00520 Unknown protein 92
VIT_14s0060g02380 Autophagy 18 ATG18d 77
VIT_06s0004g00790 Amino acid permease 40
VIT_12s0057g00900 Import inner membrane translocase subunit TIM50 85
VIT_05s0077g00700 ATP-binding 68
VIT_18s0001g12160 Unknown protein 91
VIT_05s0049g02310 Biopterin transport-related protein BT1 97
VIT_01s0011g01680 Chaperonin GroEL 53
VIT_01s0010g02480 Ankyrin repeat 87
VIT_08s0058g01110 Ovate family protein 4 OFP4 100
VIT_01s0026g02120 Microtubule end binding protein 1 (EB1) 61
VIT_08s0105g00350 DnaJ homolog. subfamily B. member 4 33
VIT_10s0003g03780 Unknown protein 45
VIT_13s0067g03730 Unknown 38
VIT_11s0016g02310 Thioredoxin 2 24
VIT_14s0006g00350 Glutamine synthetase cytosolic isozyme 2 26
VIT_08s0040g03380 Major Facilitator Superfamily 74
VIT_11s0016g02700 IAA-amino acid hydrolase 2 (ILL2) 70
VIT_05s0049g01850 Unknown protein 41
VIT_06s0009g02300 Cysteine desulfurase 23
19 VIT_10s0003g04000 ATP-dependent RNA helicase DDX51 19
VIT_07s0095g00710 Bystin 83
VIT_08s0007g05140 MAP70 protein . 99
VIT_08s0007g02220 Unknown protein 66
VIT_04s0008g06680 Unknown protein 69
VIT_07s0005g00650 U2B'' (U2 small nuclear ribonucleoprotein B) 71

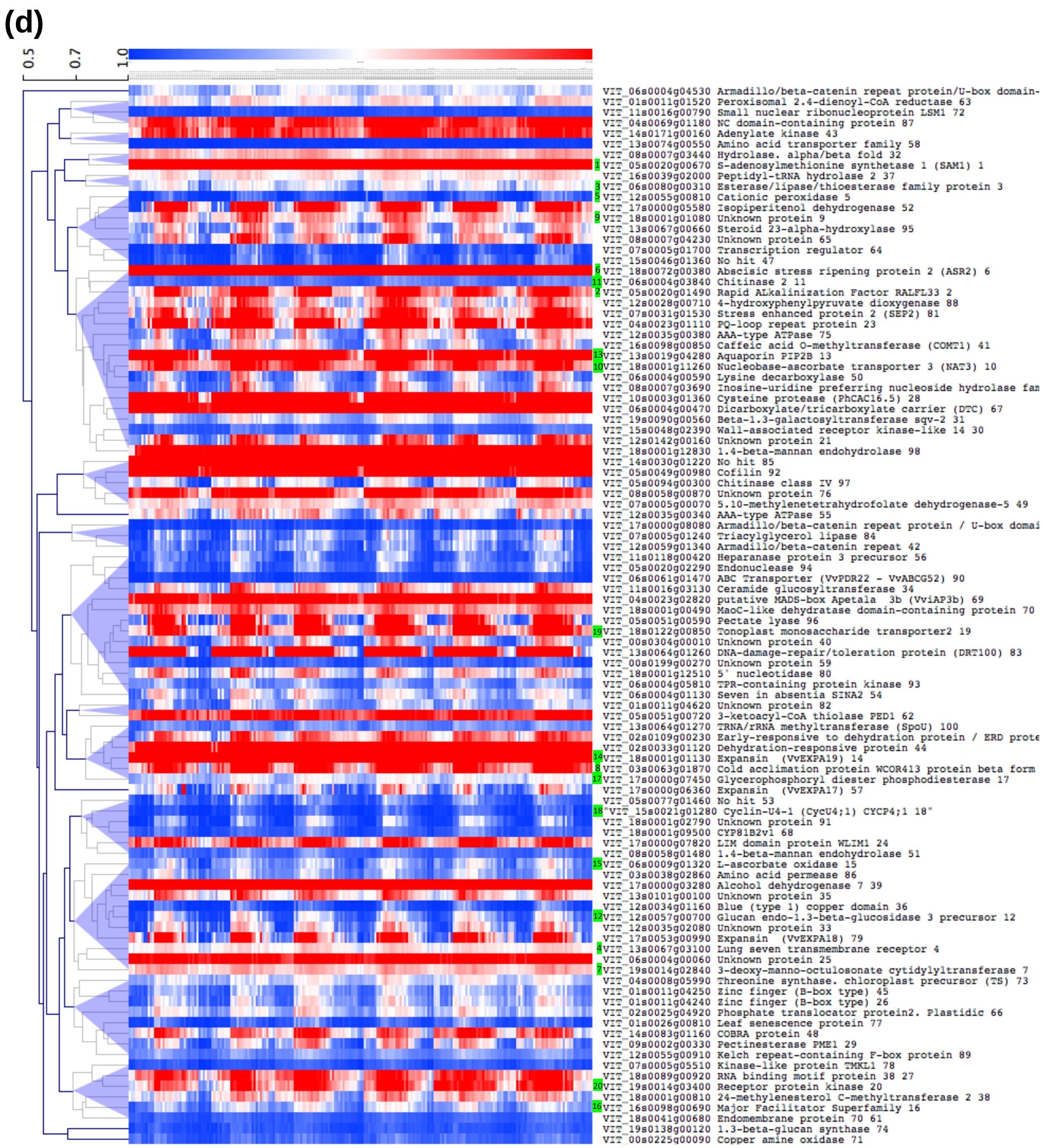

Hierarchical cluster analysis of the expression profile of the top-100 ((a) PC1 positive; (b) PC1 negative; (c) PC2 positive; (d) PC2 negative) loadings throughout berry development in PN and CS during the three vintages. The 20 genes selected to build the reduced core set-based scales are highlighted in green.

Fig. S7

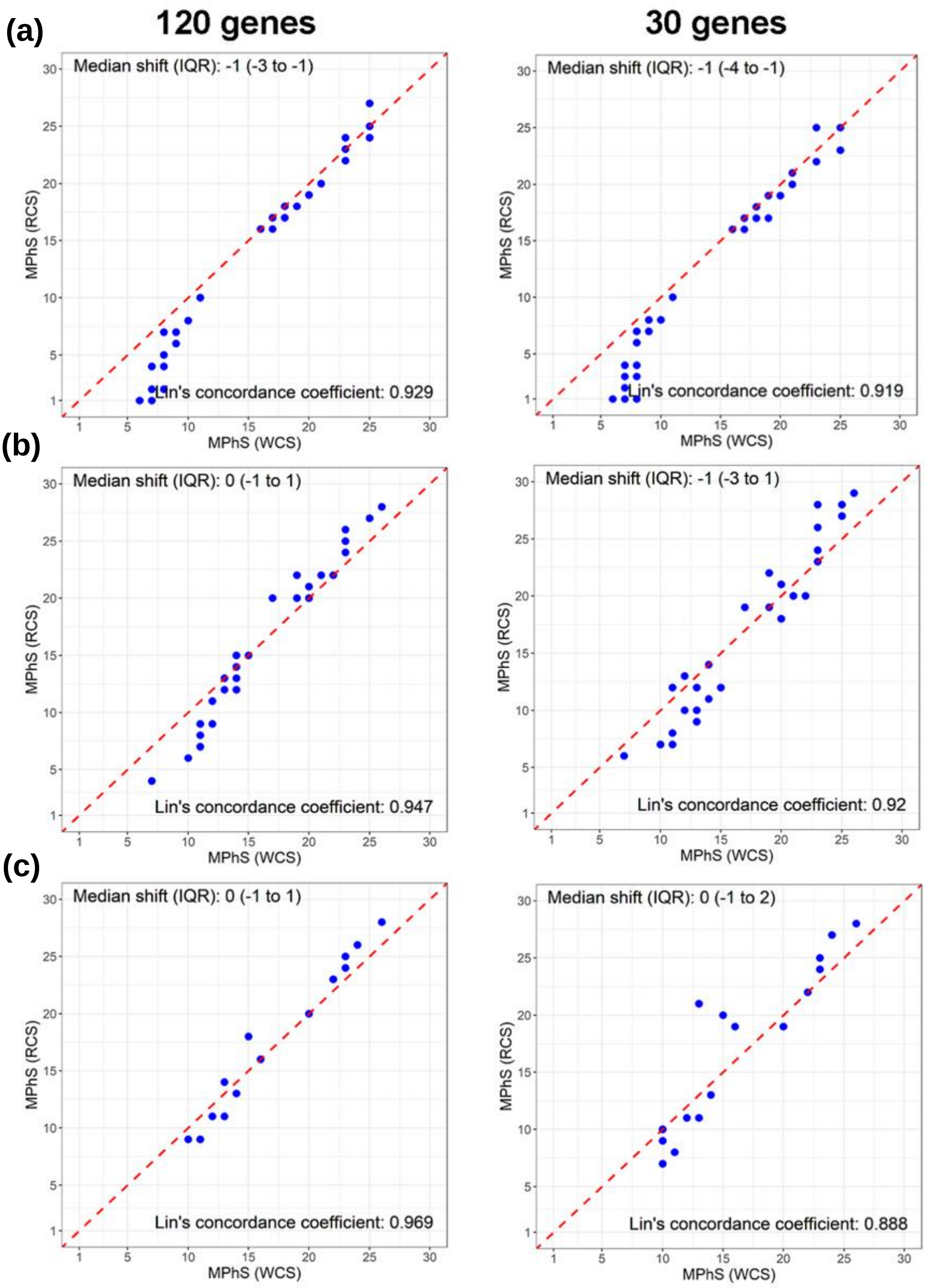

Graphical representation of the agreement between the molecular phenology scale (MPhS) calculated using entire core set of genes and the MPhS defined by reduced core sets of 120 (left) and 30 (right) genes in (a) 10 genotypes (Massonnet et al., 2017), (b) cv Corvina (Dal Santo et al., 2013) and (c) cv Garganega (Dal Santo et al., 2016). The red dashed line represents the line of perfect agreement.

The plots reports the estimated Lin's concordance coefficient and the median (with interquartile range, IQR) of the shift (difference) between the two scales.

Fig. S8

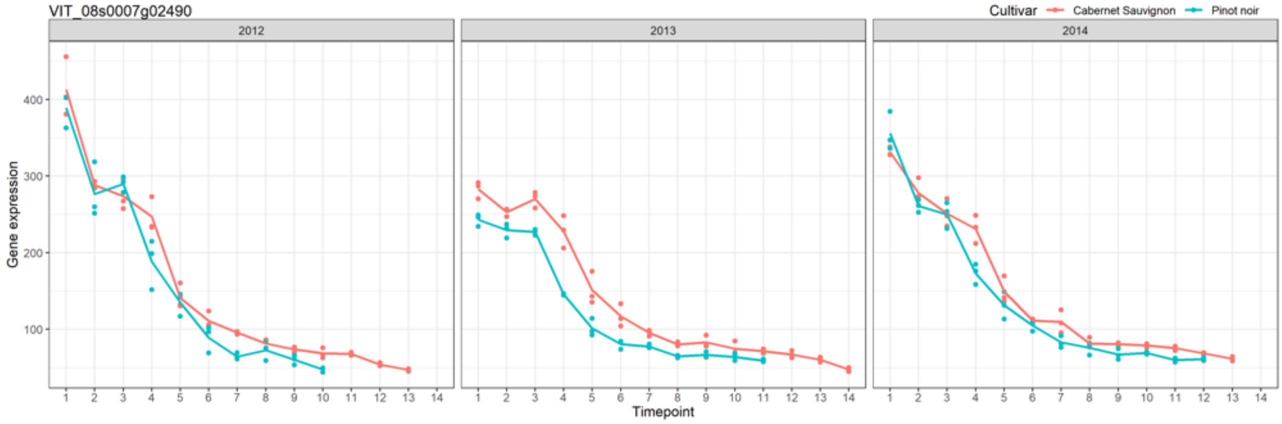

Graphical representation of the expression profile of gene VIT_08s0007g02490 over berry development in the three vintages and by cultivar, as an example. In each panel, a two-dimensional graph was plotted with the x and y axes representing the timepoint and the expression values, respectively. A line shows the pattern of the average expression m_{ji}. Three dots per timepoint represent the three replicates. Pinot noir and Cabernet Sauvignon are represented with blue and red dots and lines, respectively.

Fig. S9

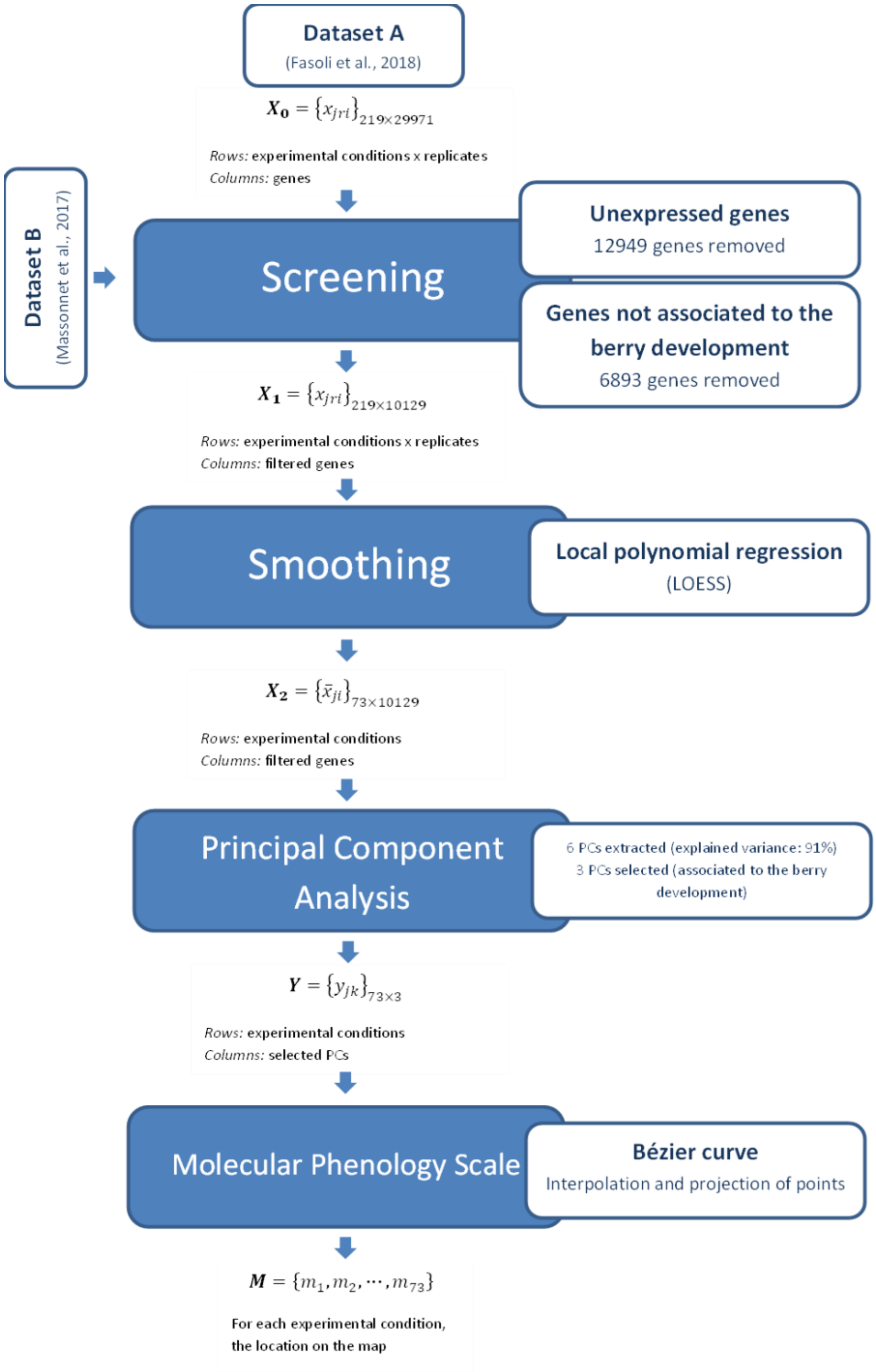

Flow chart of the pipeline.

Fig. S10

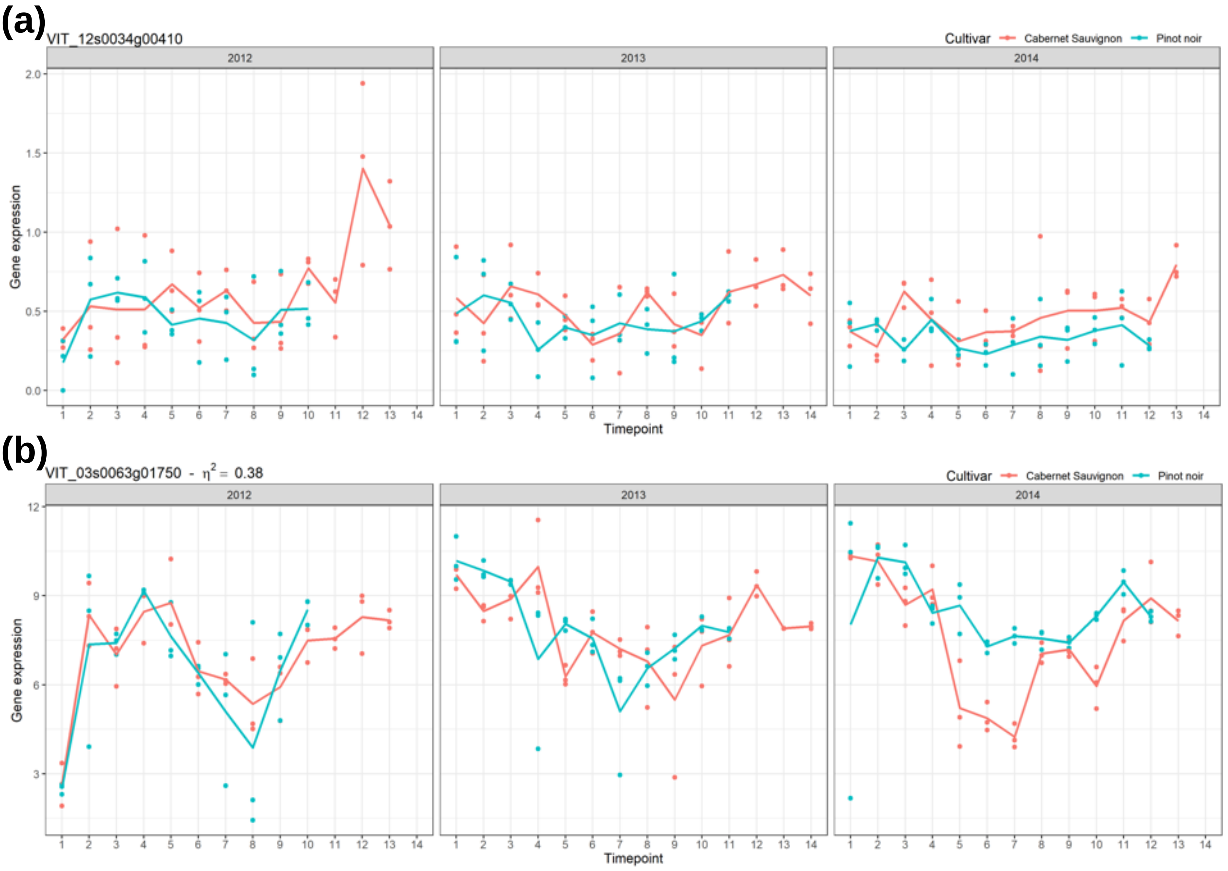

Examples of removed genes. (a) No expression (VIT_12s0034g00410). (b) Expression not associated to berry development (VIT_03s0063g01750). In each panel, a two-dimensional graph was plotted with the *x* and *y* axes representing the timepoint and the expression values, respectively. A line shows the pattern of the average expression m_{ji}. Three dots per timepoint represent the three replicates. Pinot noir and Cabernet Sauvignon are represented with blue and red dots and lines, respectively.

Fig. S11

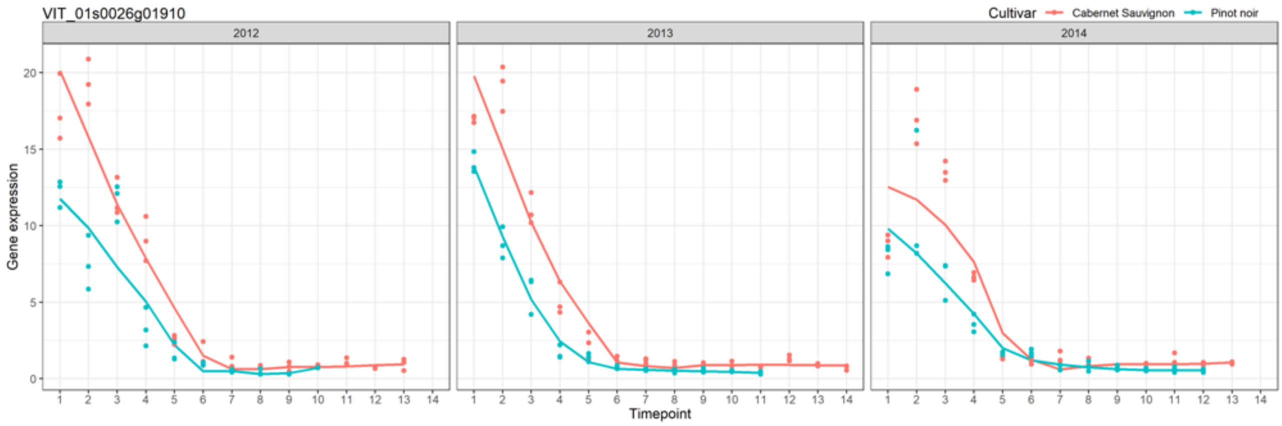

Example showing the graphical representation of smoothed expression profiles. In each panel, a two-dimensional graph was plotted with the x and y axes representing the timepoint and the expression values, respectively. A line shows the pattern of the smoothed gene expression $\bar{x}_{ji}$. Three dots per timepoint represent the three replicates. Pinot noir and Cabernet Sauvignon are represented with blue and red dots and lines, respectively.

Fig. S12

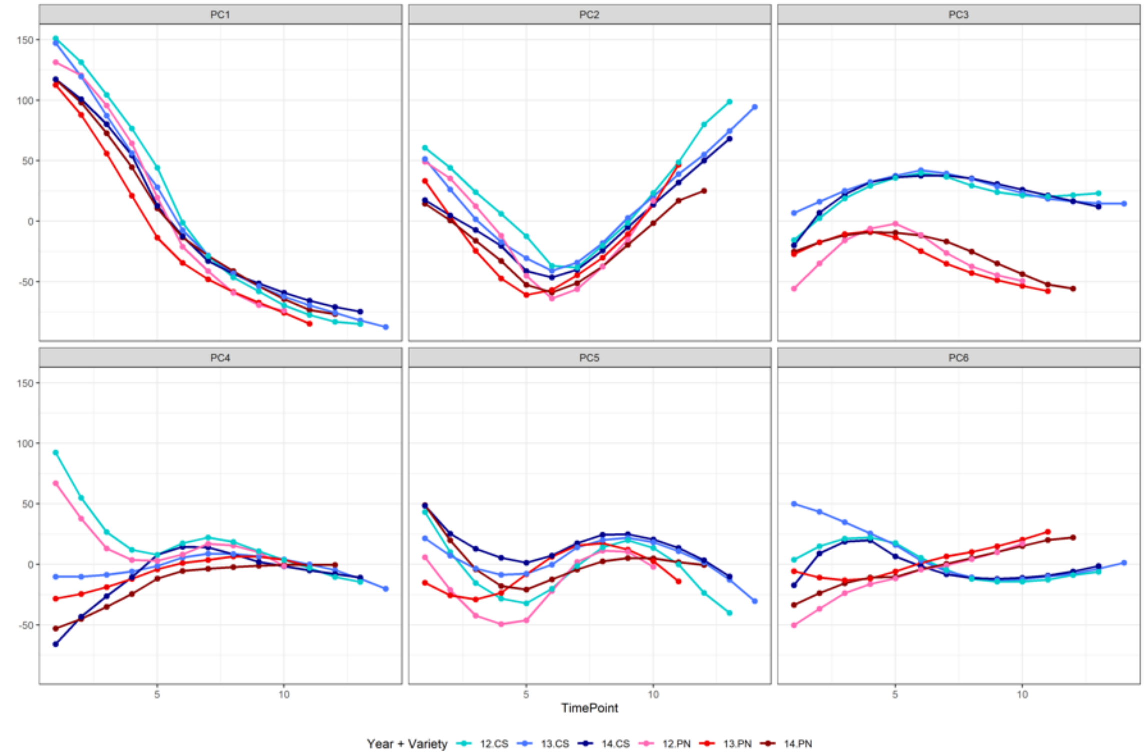

Pattern of the six PCs against the timepoints that emphasizes that the two cultivars separated by PC3 and PC6, whereas PC4 reveled vintage variability for 2012 at the early time points. PC1, PC2 and PC5 plotted over timepoints account for variation by berry development regardless cultivar and vintage.

Fig. S13

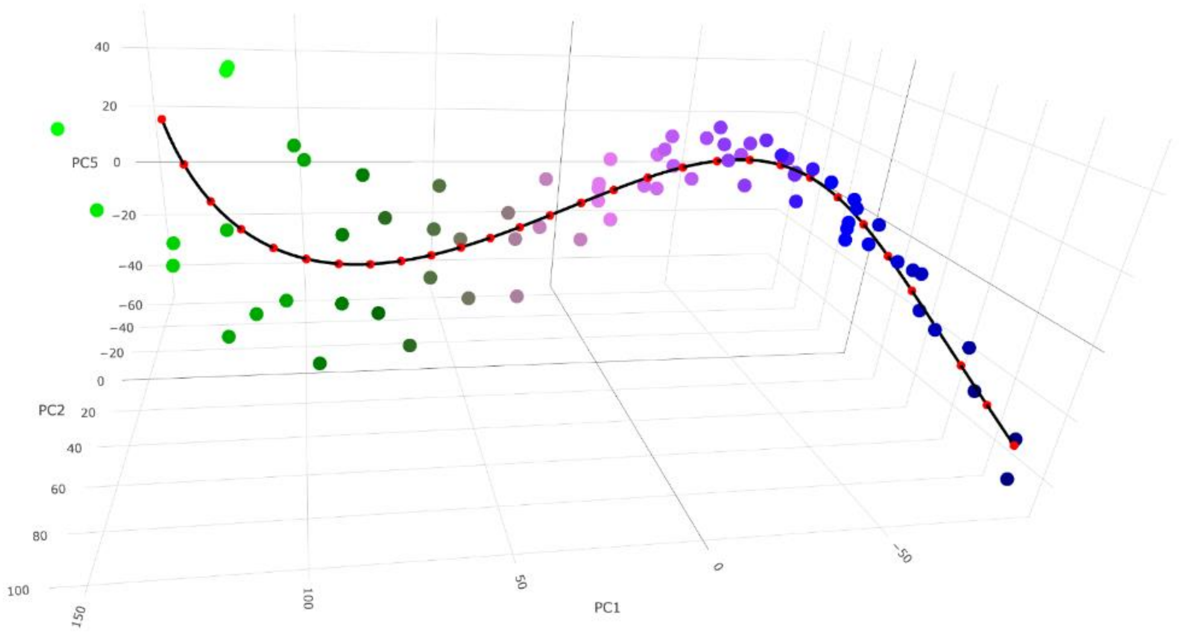

Three-dimensional scatterplot of the three selected principal components and the interpolating Bézier curve.

Dataset S1

List of the 10,129 genes selected in the whole core set (WCS).

Dataset S2

Genes highly correlated with the 20-to-30 MPhS stage progression. Spearman correlation coefficient is indicated in column D (cutoff >|0.7|). Columns E and F report the correlation coefficient of each gene also in other MPhS intervals.

Dataset S3

List of the loadings most highly correlated with Principal Components 1, 2 and 5. For each list, the correlation values and the selection of 20, 10, 5 and 2 gene subsets are reported.
